# Investigation of the Antibacterial Activity and *in vivo* Cytotoxicity of Biogenic Silver Nanoparticles as Potent Therapeutics

**DOI:** 10.3389/fbioe.2019.00239

**Published:** 2019-10-09

**Authors:** Md. Monir Hossain, Shakil Ahmed Polash, Masato Takikawa, Razib Datta Shubhra, Tanushree Saha, Zinia Islam, Sharif Hossain, Md. Ashraful Hasan, Shinji Takeoka, Satya Ranjan Sarker

**Affiliations:** ^1^Department of Biotechnology and Genetic Engineering, Jahangirnagar University, Dhaka, Bangladesh; ^2^Department of Advanced Science and Engineering, Graduate School of Advanced Science and Engineering, Waseda University, Tokyo, Japan; ^3^Department of Textile Engineering, Dhaka University of Engineering and Technology, Gazipur, Bangladesh; ^4^Department of Biochemistry and Molecular Biology, Jahangirnagar University, Dhaka, Bangladesh

**Keywords:** biogenic silver nanoparticles, antimicrobial activity, pathogenic bacteria, CellTox^**TM**^ green assay, hemocompatibility, biocompatibility, lipid peroxidation, biomarkers

## Abstract

Biogenic nanoparticles are the smartest weapons to deal with the multidrug-resistant “superbugs” because of their broad-spectrum antibacterial propensity as well as excellent biocompatibility. The aqueous biogenic silver nanoparticles (Aq-bAgNPs) and ethanolic biogenic silver nanoparticles (Et-bAgNPs) were synthesized using aqueous and ethanolic extracts of *Andrographis paniculata* stem, respectively, as reducing agents. Electron microscopic images confirmed the synthesis of almost spherical shaped biogenic silver nanoparticles (bAgNPs). The zeta potentials of the nanoparticles were negative and were −22 and −26 mV for Aq-bAgNPs and Et-bAgNPs, respectively. The antibacterial activity of bAgNPs was investigated against seven pathogenic (i.e., enteropathogenic *Escherichia coli, Salmonella typhi, Staphylococcus aureus, Vibrio cholerae, Enterococcus faecalis, Hafnia alvei, Acinetobacter baumannii*) and three nonpathogenic (i.e., *E. coli* DH5α, *E. coli* K12, and *Bacillus subtilis*) bacteria at different time points (i.e., 12, 16, 20, and 24 h) in a dose-dependent manner (i.e., 20, 40, and 60 μg) through broth dilution assay, disk diffusion assay, CellTox^TM^ Green uptake assay, and trypan blue dye exclusion assay. The lowest minimum inhibitory concentration value for both the bAgNPs was 0.125 μg. Et-bAgNPs showed the highest antibacterial activity against *S. aureus* at 60 μg after 16 h and the diameter of inhibited zone was 28 mm. Lipid peroxidation assay using all the bacterial strains revealed the formation of malondialdehyde–thiobarbituric acid adduct due to the oxidation of cell membrane fatty acids by bAgNPs. The bAgNPs showed excellent hemocompatibility against human as well as rat red blood cells. Furthermore, there was no significant toxicity observed when the levels of rat serum ALT, AST, γ-GT (i.e., liver function biomarkers), and creatinine (i.e., kidney function biomarker) were determined.

## Introduction

Pathogenic bacteria are the real threat to mankind due to their ability to develop resistance mechanism against the commercially available antibiotics (Aslam et al., 2018). Most of the patients in the post-operative care get infected through multidrug-resistant bacteria (e.g., methicillin-resistant *Staphylococcus aureus* and multidrug-resistant *Salmonella typhimurium*) (Khan et al., 2011; Blair et al., 2015; Ramalingam et al., 2016). Frequent use and abuse of antibiotics have led to the evolution of multidrug-resistant bacteria, also called “superbugs,” which are resistant to all the commercially available antibiotics (Wang et al., 2017). Inherent mutations and lateral gene transfer among bacteria have also been contributing to the emergence of superbugs (Wang et al., 2017). On the other hand, the effectiveness of antibiotics remains for a short period of time and, therefore, used for continuous antibacterial treatment (Han et al., 2019). Therefore, it is crucial to develop new antibacterial agents with the ability to kill any pathogenic bacteria and superbugs and with prolonged and broad-spectrum antibacterial activity.

Inorganic nanoparticles including gold (Au) (Ramamurthy et al., 2013; Vijayakumar et al., 2017b), silver (Ag) (Lok et al., 2007; Ramamurthy et al., 2013; Shanthi et al., 2016; Thaya et al., 2016; Malaikozhundan et al., 2017b), graphene oxide (GO) (Rasool et al., 2016), zinc oxide (ZnO) nanoparticles (NPs) (Lu et al., 2008; Malaikozhundan et al., 2017a; Vijayakumar et al., 2017a) have been widely explored as antibacterial agents because of their excellent physical and chemical properties. Among them, silver nanoparticles (AgNPs) have been widely used in pharmaceuticals and cosmetics and in the treatment of water because of their antibacterial propensity against a wide range of bacteria (Ramalingam et al., 2016). However, the chemically synthesized AgNPs are highly unstable and get aggregated because they are prone to easy oxidation (Gondikas et al., 2012; Levard et al., 2012). The aggregated AgNPs lose their antibacterial activity (Gondikas et al., 2012; Levard et al., 2012), and the leakage of Ag^+^ from the chemically synthesized AgNPs creates health as well as environmental hazard (Ramalingam et al., 2016). In addition, the chemical synthesis of AgNPs is tedious, expensive, and hazardous for the environment. Recent approaches for the synthesis of AgNPs using plant extract as the source of reducing agents instead of chemicals including NaHB_4_ (Mehr et al., 2015) and citric acid (Kilin et al., 2008) for the reduction of silver nitrate are easier, more effective, cost-effective, and ecofriendly (Schluesener and Schluesener, 2013; Poulose et al., 2014). There are several plants including *Andrographis paniculata, Phyllanthus emblica*, and *Centella asiatica* have medicinal applications (Mondal et al., 2017; Polash et al., 2017; Masum et al., 2019). Because these plants contain various secondary metabolites (e.g., alkaloids, phenols, flavonoids, terpenoids, tannins, andrographolides, and so on), they have antimicrobial, antioxidant, anticancer, and antidiabetic activities (Jain et al., 2019). The reducing potential of the plant extracts is used to reduce AgNO_3_ into AgNPs (He et al., 2016), which are frequently used for their anti-inflammatory, antimicrobial, antifungal, anti-angiogenesis, antiplatelet (He et al., 2016), and anticancer activities (Tolaymat et al., 2010; Dar et al., 2013).

Silver nanoparticles are not toxic for the human healthy peripheral lymphocytes (Gengan et al., 2013). However, they have high affinity and significant toxicity to bacteria as well as cancer cells (Greulich et al., 2012; Gengan et al., 2013). Recently, AgNPs have been used in cancer theranostics (i.e., diagnosis and treatment) (Ong et al., 2013). They are used as drugs by themselves or targeted delivery vehicles of anticancer drugs or probes for the early detection of cancer (Wei et al., 2012; Locatelli et al., 2014).

In this study, we synthesized two different types of biogenic silver nanoparticles (i.e., Aq-bAgNPs and Et-bAgNPs) using aqueous and ethanolic extract of *A. paniculata* stem. The as-synthesized bAgNPs were characterized by UV-Vis spectroscopy, Fourier transform infrared (FTIR) spectroscopy, energy dispersive X-ray spectroscopy (EDS), powder X-ray diffractometer (XRD), transmission electron microscopy (TEM), scanning electron microscopy (SEM), and a scanning TEM unit with a high-angle annular dark field (HAADF) detector. The hydrodynamic size and zeta potential of the as-synthesized bAgNPs were determined by zeta size analyzer. The antibacterial potential of bAgNPs was investigated against seven pathogenic bacterial strains including enteropathogenic *E. coli* (EPEC), *Salmonella typhi, S. aureus, Vibrio cholerae, Enterococcus faecalis, Hafnia alvei*, and *Acinetobacter baumannii*. Food- and water-borne bacteria are the cause of various acute or chronic infections including diarrhea, cholera, salmonellosis, shigellosis, traveler's diarrhea, typhoid fever, pneumonia, and dysentery (Panayidou et al., 2014). Therefore, the pathogenic bacterial strains were used to investigate the antibacterial propensity of our as-synthesized bAgNPs. In addition, three nonpathogenic bacterial strains such as *E. coli* DH5α, *E. coli* K12, and *Bacillus subtilis* RBW were also used in the experiment. The antibacterial potential of bAgNPs was investigated through determination of the diameter (in millimeter) of the zone of inhibition (ZOI) through disk diffusion assay, determination of minimum inhibitory concentration (MIC) value through broth dilution method, CellTox^TM^ green assay, and trypan blue dye exclusion assay. Lipid peroxidation (LPO) assay was performed to investigate the mechanism of antibacterial propensity of bAgNPs. Furthermore, we have investigated the hemocompatibility of bAgNPs against human as well as rat red blood cells (RBCs). The biocompatibility of bAgNPs was investigated *in vivo* using Wister rat model. The effect of bAgNPs on liver and kidneys was investigated through determining the level of serum ALT, AST, and γ-GT as liver function biomarkers and serum creatinine as kidney function biomarker.

## Materials and Methods

### Materials

Silver nitrate (AgNO_3_), potassium bromide (KBr), EDTA, and absolute ethanol were purchased from Sigma-Aldrich (USA). CellTox^TM^ Green dye was purchased from Promega (USA). Trypan blue dye was collected from Alfa Aesar (United Kingdom). Agar powder was purchased from Titan Biotech Ltd. (India). Peptone, yeast extract, and sodium chloride were collected from Unichem (China). Trichloroacetic acid (TCA) and thiobarbituric acid (TBA) were purchased from Merck (Germany) and JT Baker (USA), respectively. The biochemical analysis kits for serum AST, ALT, and γ-GT analysis were purchased from Vitro Scient (Egypt) and the serum creatinine determination kit was purchased from Crescent Diagnostics (The Kingdom of Saudi Arabia). Seven pathogenic [i.e., enteropathogenic *Escherichia coli* (ATCC 43887), *S. typhi* (ATCC 14028), *S. aureus* (ATCC 25923), *V. cholerae* (ATCC 55057), *E. faecalis* (ATCC 29212), *H. alvei* (ATCC 51815), and *A. baumannii* (ATCC 17978)] and three nonpathogenic [i.e., *E. coli* DH5α (ATCC 25922), *E. coli* K12 (ATCC 10798), and *B. subtilis* RBW (ATCC 6051)] strains were obtained from the Department of Biotechnology and Genetic Engineering, Jahangirnagar University, Savar, Dhaka 1342, Bangladesh. Thirty Wistar male rats (weighing between 170 and 180 g) were obtained from the Department of Biochemistry and Molecular Biology, Jahangirnagar University, Dhaka, Bangladesh.

### Methods

#### Synthesis of Biogenic Silver Nanoparticles (bAgNPs)

*A. paniculata* stem extract was used to prepare biogenic silver nanoparticles (bAgNPs) since it is cost effective and has medicinal property. The fresh stem of *A. paniculata* was collected and pulverized, and 10% aqueous as well as 10% ethanolic extracts were prepared according to our previously published protocol (Polash et al., 2017). Briefly, 10 mM AgNO_3_ solution was prepared and mixed with the as-prepared aqueous as well as ethanolic stem extracts separately at a ratio of 9:1 (i.e., 36 ml AgNO_3_ and 4 ml plant extracts). The mixture of AgNO_3_ solution and the extracts was incubated at 30°C for 24 h upon constant stirring in a dark chamber to reduce the photo activation of AgNO_3._ Herein, the plant extract was used as a reducing agent and the reduction of Ag^+^ to Ag^0^ was confirmed by the change of color of the solution from colorless to brown (Ahmed et al., 2016). The reaction mixture was then centrifuged at 16,873 g for 1 h at room temperature to remove the unconjugated plant extract and AgNO_3_. The supernatant was aspirated and the bAgNPs generated by aqueous as well as ethanolic extracts were deposited as pellet at the bottom of the tube. Finally, the bAgNPs were washed twice with distilled water and redispersed in distilled water.

#### Characterization of Biogenic Silver Nanoparticles (bAgNPs)

The as-synthesized biogenic silver nanoparticles (bAgNPs) were characterized using a UV-Vis spectrophotometer (Specord® 205, Analytik Jena, Germany) and FTIR spectroscopy (IRPrestige-21, SHIMADZU, Japan). The hydrodynamic size and zeta potential of bAgNPs were determined with a zeta size analyzer (Nano-ZS90; Spectris PLC, Egham, England) after sonicating for 30 min in a bath-type sonicator. The shape, morphology, and composition of biogenic nanoparticles were analyzed with TEM with a field emission gun (HF-2200; Hitachi, Tokyo, Japan) coupled with energy dispersive X-ray spectroscopy (EDAX Genesis; AMETEK, Pennsylvania, USA) operating at 200 kV. A scanning TEM unit with a HAADF detector and secondary electron (SE) detector were also equipped. The nanoparticles were neither stained with any conventional staining agent nor coated with any conductive metal before observing them under electron microscopy.

The powder X-ray diffraction (XRD) patterns of bAgNPs were obtained using a powder X-ray diffractometer (GNR X-Ray Explorer, Italy) equipped with Cu Kα (0.154 nm) at 25 mA without Ni filter. The XRD spectrum was obtained by scanning the diffraction angle (2θ) region at 30 kV. The diffraction angle was varied in the range of 10–50° and the scanning rate was 5°/s.

#### Antimicrobial Activity Assay

The antimicrobial activity of the bAgNPs was investigated against seven pathogenic (i.e., Enteropathogenic *E. coli, S. typhi, S. aureus, V. cholerae, E. faecalis, H. alvei*, and *Acinetobacter*) and three nonpathogenic (i.e., *E. coli* DH5α, *E. coli* K12, and *B. subtilis*) bacterial strains. Among all the bacteria, *S. aureus, E. faecalis*, and *B. subtilis* are Gram-positive, and the remaining bacterial strains are gram negative. The antibacterial activity of bAgNPs was determined in terms of their MIC value, ZOI in millimeters, trypan blue dye exclusion assay, and CellTox^TM^ Green cytotoxicity assay.

##### Determination of the MIC value

The MIC value was determined to identify the minimum amount of bAgNPs (i.e., Aq-bAgNPs and Et-bAgNPs) required to inhibit the growth of a particular bacterial strain. The MIC value of the as-synthesized bAgNPs was determined according to the broth dilution method (Mondal et al., 2017). Briefly, 990 μl of fresh LB broth was inoculated with 10 μl of overnight grown bacterial culture and incubated at 37°C and 120 rpm for 4 h. After incubation, different amounts (i.e., 0.125, 0.25, 0.5, 1, 2, 3, 4, 5, and 6 μg) of either Aq-bAgNPs or Et-bAgNPs was added to the bacterial culture and incubated at 37°C and 120 rpm overnight. The optical density (OD) of the respective bacterial culture was then measured at 600 nm using UV-Vis spectrophotometer (Optizen, POP, Korea) to determine the MIC values of bAgNPs.

##### Determination of ZOI

The antimicrobial activity of the as-synthesized Aq-bAgNPs and Et-bAgNPs was determined by disk diffusion method (Polash et al., 2017). Briefly, all the tested bacterial strains were cultured in Luria Bertani (LB) medium at 37°C and 120 rpm overnight. From the respective bacterial culture, 100 μl was spreaded consistently on LB agar plates. Metrical filter paper disks containing different amounts of bAgNPs (i.e., 60, 40, and 20 μg) were placed on the LB agar plates containing uniformly spreaded bacteria. The LB agar plates were then incubated overnight at 37°C for optimum growth of bacteria. The antibacterial activity of Aq-bAgNPs and Et-bAgNPs was determined by the presence of clear zones surrounding the disks that were different with different amounts of bAgNPs (i.e., Aq-bAgNPs and Et-bAgNPs) and confirm the inhibition of the growth of bacteria. After incubation, the diameter of the clear zones was measured using slide calipers at different time points such as 12, 16, 20, and 24 h.

##### Trypan blue dye exclusion assay

Trypan blue is a negatively charged dye and can't pass through the intact cell wall of bacteria. Therefore, it is used to differentiate cell wall compromised dead bacteria from cell wall intact live bacteria. This is because the dead cells allow the uptake of trypan blue dye through their damaged cell membrane (Tran et al., 2011; Ranjan Sarker et al., 2019). The trypan blue dye exclusion assay was performed according to our previously established protocol with little modifications (Ranjan Sarker et al., 2019). Briefly, 20 μl (1 μg/μl) of Aq-bAgNPs and Et-bAgNPs was mixed separately with 80 μl of overnight grown culture (1 × 10^6^ CFU/ml) of the respective bacterial strains (i.e., seven pathogenic and three nonpathogenic bacterial strains) and incubated at 37°C and 120 rpm for 1.5 h. The bacterial cultures treated with bAgNPs were mixed with 0.4% trypan blue solution at a ratio of 1:1 and incubated at room temperature for 15 min before taking images of live and dead bacterial cells using a phase contrast microscope (Olympus BX50 Fluorescence Microscope, Olympus, Japan) at 40× magnification.

##### CellTox^***TM***^ green assay

CellTox^TM^ Green is a fluorescent dye that penetrates through the disrupted cell membrane and emits green fluorescence upon binding with the DNA (Zhang et al., 1999). Overnight grown bacterial culture was diluted with LB broth and the concentration was set to 1 × 10^7^ CFU/ml. Either Aq-bAgNPs or Et-bAgNPs (20 μl; 1 μg/μl) were mixed with 80 μl (1 × 10^7^ CFU/ml) of the respective bacterial strains. Then, 900 μl of fresh LB broth was added to bAgNPs containing bacterial culture before incubating at 37°C and 120 rpm for 2 h. After the incubation, 1 μl of CellTox^TM^ Green reagent (2 ×) was mixed properly with bAgNPs containing bacterial culture and incubated at room temperature for another 30 min in the dark. Twenty microliters of the CellTox^TM^-treated bacterial culture was kept aside to observe the dead cells under fluorescence microscope (Olympus BX50 Fluorescence Microscope, Olympus, Japan). The remaining bacterial suspension was used to measure the fluorescence intensity of the green fluorescent dye treated bacterial cultures using a spectrofluorophotometer (SHIMADZU RF-6000, Japan) at 490 nm. Biogenic AgNPs with control groups (i.e., cells and media) were run without CellTox^TM^ Green alongside the treatment groups. All experiments were carried out in triplicate.

#### LPO Assay

The LPO potential of bAgNPs was investigated according to previously established protocol (Singh et al., 2009; Ranjan Sarker et al., 2019). Briefly, 1 ml of each of the bacterial cultures was mixed separately with 200 μl (i.e., 200 μg) of each of the bAgNPs and incubated for 30 min at room temperature. After centrifugation, 2 ml of 10% TCA was mixed with the bAgNP-treated bacterial culture and centrifuged at 10,416 g for 35 min at room temperature to separate the insoluble cellular components. The supernatant was taken out and centrifuged again in the same relative centrifugal force (*g*) for 20 min to remove any protein precipitates as well as dead cells. Finally, the supernatant containing malondialdehyde was collected in a fresh tube and mixed with freshly prepared 4 ml of 0.67% TBA solution and incubated in a hot water bath for 10 min to facilitate the formation of malondialdehyde-TBA adduct before cooling down to room temperature. The absorbance of the malondialdehyde-TBA adduct was measured by UV-Vis spectrophotometer (Specord® 205, Analytik Jena, Germany) at 532 nm.

#### Hemocompatibility Assay

The hemocompatibility of bAgNPs (i.e., Aq-bAgNPs, and Et-bAgNPs) with human as well as rat RBCs was investigated according to previously established protocol with some modification (Li et al., 2014; Ranjan Sarker et al., 2019). Briefly, 6 ml of human blood was collected from the left hand of donor through venipuncture method and collected in a vacutainer blood tube containing 10% EDTA. On the other hand, two rats were anesthetized with 0.3 ml/250 g ketamine/xylazine (100 mg/ml ketamine + 20 mg/ml xylazine) before the collection of blood and ~6 ml (3 ml from each rat) of blood was drawn from the inferior vena cava and collected in a vacutainer blood tube containing 10% EDTA. RBCs of human as well as rat were separated from serum through centrifugation at 500 g for 10 min at room temperature. The serum was then discarded and RBCs were resuspended in 5 ml of phosphate buffered saline (PBS) and centrifuged again at 500 g for 10 min. The cell suspension was washed twice with 150 mM NaCl solution at 3000 g for 3 min. Finally, 0.1 ml of RBCs solution was mixed with 0.4 ml of different amounts of bAgNPs (i.e., 10, 20, 30, 40, 50, 60, 80, 100, 200, 400, 800, and 1,600 μg) and incubated at 37°C and 150 rpm for 30 min. After incubation, the RBCs and bAgNPs mixtures were centrifuged at 1,377 g for 5 min at room temperature. The supernatant was taken out and the absorbance was measured at 570 nm. Herein, RBCs incubated with PBS and water were considered as negative and positive controls, respectively (Li et al., 2014). All the samples were taken in triplicate. The percentage of hemolysis was calculated using the following formula:

(1) $$\begin{array}{r} \%\;of\;hemolysis=\left(\frac{\begin{array}{c} Absorbance\;of\;sample\;-\\ Absorbance\;of\;negative\;control \end{array}}{\begin{array}{c} Absorbance\;of\;positive\;control\;-\\ Absornbance\;of\;negative\;control \end{array}}\right)\times \;100 \end{array}$$

#### *In vivo* Cytotoxicity Assay

##### Intravenous delivery of bAgNPs

Wistar male rats were used for the study and 30 rats were divided into five groups (*n* = 6). The rats were housed in a hygienic environment under controlled temperature (23 ± 2°C) and humidity (55 ± 7%) with a 12-h day and 12-h night cycle (Lee et al., 2018). The rats were allowed to acclimatize for a week before the delivery of bAgNPs. According to Katarzyna et al., if the size of AgNPs is ~20 nm, their deposition is higher in the tissues (e.g., liver, spleen, kidney, and brain) when compared to that of the larger nanoparticles (Dziendzikowska et al., 2012; Yang et al., 2017). Since the size of our as-synthesized Aq-bAgNPs and Et-bAgNPs are 24.90 and 25.24, respectively, we fixed the highest dose as 5 mg/kg of rat body weight. All the rats were anesthetized with 0.3 ml/250 g ketamine/xylazine (100 mg/ml ketamine + 20 mg/ml xylazine) before intravenous delivery of bAgNPs. Two doses (i.e., 2 and 5 mg/kg of rat body weight) of both Aq-bAgNPs and Et-bAgNPs were delivered through intravenous route via tail's vein. After the delivery of bAgNPs, the rats were kept in the cage under the same condition for 7 days. After 7 days, blood (~3 ml) was drawn from all the rats and centrifuged at 538 g for 10 min at room temperature to separate the serum. The serum samples were stored at −20°C for further biochemical analysis. The experiments in this study were approved by the Biosafety, Biosecurity & Ethical Committee of Jahangirnagar University (BBEC, JU/M 2019(4)1).

##### Investigation of liver and kidney function biomarkers

To determine whether bAgNPs have any toxic effects to rat tissues, we measured the concentration of several enzymes whose level usually get hiked once there is any damage in the liver and kidneys. We measured the level of serum aspartate aminotransferase (AST), alanine aminotransferase (ALT), gamma-glutamyltransferase (γ-GT), and creatinine of the experimental rats and compared them with that of control rats. The serum AST, ALT, and γ-GT are known as the liver function biomarkers and creatinine is known as the kidney function biomarker. The concentrations of all the biomarkers were measured according to the manual provided with the reagent kits, and the detailed protocol has been added in the supplementary section.

### Statistical Analysis

All the data were presented as the mean ± SEM. Data were subjected to statistical analysis using one-way ANOVA with GraphPad Prism 5.0 (GraphPad Software Inc., San Diego, CA). *Post hoc* test was performed using the Dunnett test. The mean values were considered to be statistically significant at *P* < 0.05 (Adeyemi and Adewumi, 2014). All experiments were carried out in triplicate, and the results presented are the average measurements of the runs with standard deviation.

## Results and Discussion

### Characterization of bAgNPs

**Figure 1:**
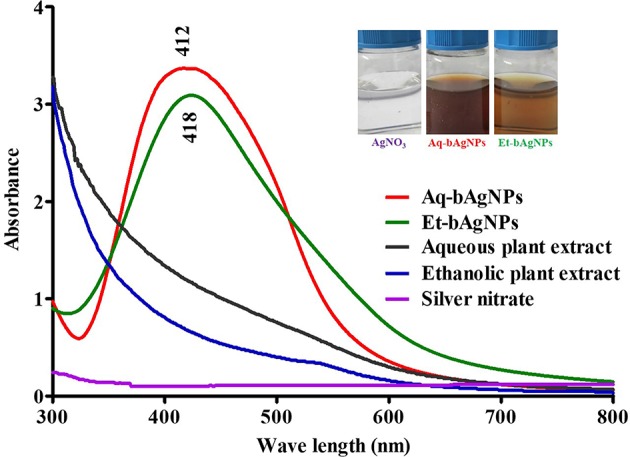
UV-Vis spectra of biogenic silver nanoparticles (bAgNPs).

The colorless AgNO_3_ (10 mM) solution turned brown as soon as *A. paniculata* stem extract was added into it and incubated for ~24 h (Figure 1). When aqueous stem extract was added into AgNO_3_ solution, the color of the solution turned dark brown when compared to that of brown in the case of ethanolic stem extract. The change of color confirms the reduction of Ag^1+^ to Ag^0^ due to the presence of secondary metabolites including flavonoids and different types of andrographolides in *A. paniculata* stem extract (Singh et al., 2018). Flavonoids and andrographolides present in the *A. paniculata* stem extract would bind to the biogenic nanoparticles surface as capping agents. The final yield of both the bAgNPs was 9.1%. The UV-Vis spectra confirm the synthesis of biogenic silver nanoparticles (bAgNPs) because the absorption maximum (λ_max_) for Aq-bAgNPs and Et-AgNPs is ~412 and ~418 nm (Figure 1), respectively, which are almost equal to the surface plasma resonance (SPR) value of AgNPs (i.e., 420 nm) (Ramalingam et al., 2016). At a particular wavelength, electrons on the metal surface undergo a collective oscillation when excited by light resulting in the strong scattering and absorption properties of bAgNPs (Li et al., 2010). Et-bAgNPs derived from the ethanolic extract of *A. paniculata* stem appeared brown due to the presence of higher amount of andrographolides in the ethanolic stem extract that caused greater reduction of Ag^1+^ at room temperature (Daniel et al., 2013). Therefore, a slight red shift was observed in case of Et-bAgNPs.

The analysis of FTIR spectra confirm the presence of various bioactive compounds having different functional groups in the plant extract as well as in the bAgNPs (Alshaye et al., 2017; Elamawi et al., 2018). The spectra of Aq-bAgNPs (Figure S1) and aqueous stem extract of *A. paniculata* (Figure S1) revealed strong bands at (839 and 797), (1,048 and 1,043), (1,376 and 1,398), (1,615 and 1,650), (2,915 and 2,923), and (3,342 and 3,377 cm^−1^) that were attributed to aromatic C–H (aryl group), C–OH stretching, C–N stretching, carbonyl stretch, C–H asymmetric stretching, and N–H stretching, respectively (Basseter and Silverstein, 1992). The spectra of Et-bAgNPs (Figure S1) and ethanolic stem extract of *A. paniculata* (Figure S1) showed strong bands at (804 and 797), (1,034 and 1,055), (1,383 and 1,394), (1,610 and 1,655), (2,916 and 2,930), and (3,321 and 3,398 cm^−1^) that were due to aromatic C–H (aryl group), C–OH stretching, C–N stretching, carbonyl stretch, C–H asymmetric stretching, and N–H stretching, respectively (Ahmed et al., 2016). The intense band at 1,370 cm^−1^ is characteristic of silver nitrate (Rogachev et al., 2013). The FTIR spectra clearly showed that the hydroxyl group (-OH) and carboxyl group (-COOH) containing bioactive compounds present in *A. paniculata* stem extracts act as capping agents in the synthesis of bAgNPs. The observed peaks were mainly attributed to flavonoids and andrographolides present in the plant extract (Banerjee et al., 2014).

The average hydrodynamic diameter of both bAgNPs was larger than that of their nominal particle size (Table S1). The hydrodynamic diameters of Aq-bAgNPs and Et-bAgNPs were 428.2 ± 197.0 and 190.1 ± 102 nm, respectively. The polydispersity index (PDI) of both the bAgNPs was almost the same (i.e., 0.4). The zeta potentials of Aq-bAgNPs and Et-bAgNPs were −22.1 ± 0.9 and −26.1 ± 1.4 mV, respectively (Table S1). The negative zeta potential of bAgNPs is due to the adsorption of bioactive molecules onto the particle surface (Rao and Paria, 2015).

**Figure 2:**
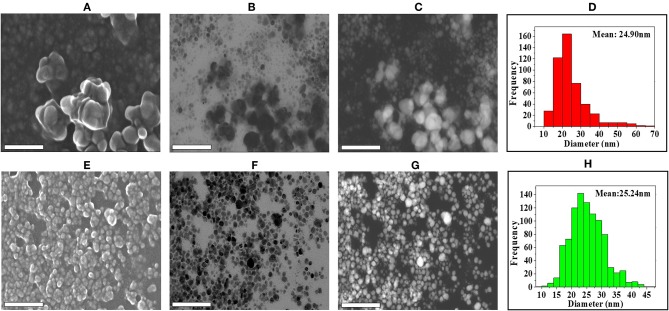
Analysis of the shape, morphology, and size of biogenic Ag nanoparticles (bAgNPs) with scanning transmission electron microscope (STEM). SE images show the formation of Aq-bAgNPs **(A)**. Scanning TEM images **(B)**, high-angle annular dark-field (HAADF) images (Z-contrast images) **(C)**, and histogram for particle size distribution **(D)** show the formation, spherical shape, and size of Aq-bAgNPs. Similarly, SE **(E)**, scanning TEM **(F)**, HAADF images **(G)**, and histogram for particle size distribution **(H)** show the formation spherical shape and size of Et-bAgNPs. Scale bar: 200 nm.

**Figure 3:**
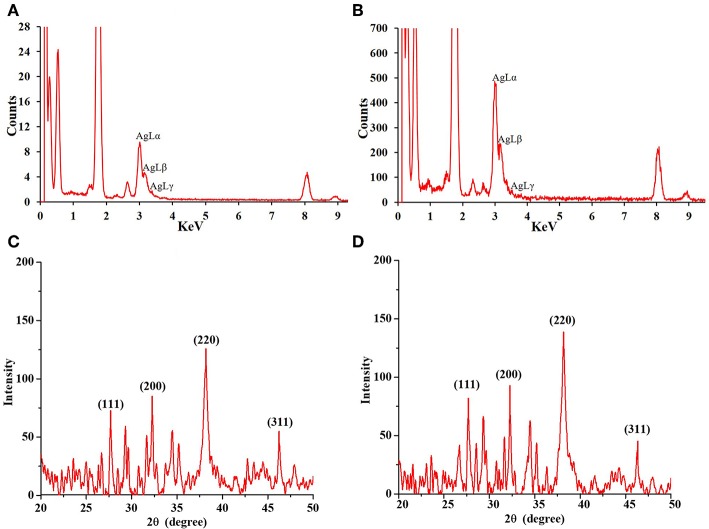
Energy dispersive X-ray (EDS) spectra of Aq-bAgNPs **(A)**, and Et-bAgNPs **(B)**. Peaks at ~3 keV indicate the presence of Ag in the biogenic nanoparticles. Powder XRD analysis of as-synthesized Aq-bAgNPs **(C)**, and Et-bAgNPs **(D)**.

The shape and morphology of biogenic nanoparticles were analyzed using scanning transmission electron microscope (STEM). For both the bAgNPs, STEM images showed that Ag nanoparticles were surrounded with polymers and were not directly observed. In addition, respective TEM or HAADF images also exhibited that Ag nanoparticles were coated with polymers and most of the nanoparticles were spherical in shape (Figure 2). The particle size distribution (PSD) graphs (i.e., histogram) were drawn from the TEM images of both Aq-bAgNPs and Et-bAgNPs (Figures 2D,H). The average size of Aq-bAgNPs and Et-bAgNPs was 24.90 and 25.24 nm, respectively. The hydrodynamic size of bAgNPs was larger than the size of the same nanoparticles observed under TEM. This is because TEM measures the size of the dried nanoparticles. On the other hand, DLS measures the hydrodynamic size of the particles containing conjugated/adsorbed polymers on the surface. The stability of the nanoparticles depend on the nature of the conjugated polymers as well as the physicochemical properties of solvents used to disperse them (Domingos et al., 2009). If the particles have the tendency to agglomerate, the hydrodynamic size is usually larger. EDS analysis confirmed that nanoparticles are composed of Ag, C, and O (Figures 3A,B). The characteristic Ag peak was observed at ~3 keV. Other peaks, such as Cu and Si, are artifacts used during the analysis by TEM grid and silicon oil for a vacuum pump, respectively. Considering these results in EM observation and EDS analysis, we judged that Ag nanoparticles were coated with bioactive polymers present in *A. paniculata* stem extracts.

Powder XRD analysis was performed to identify the phase, orientation, and grain size of the as-synthesized bAgNPs (i.e., Aq-bAgNPs and Et-bAgNPs). Figures 3C,D clearly show the characteristic diffraction peaks associated with the crystalline silver. The Braggs peaks at 2θ degrees were observed at 27.68°, 32.27°, 38.16°, and 46.27° which correspond to the Miller indices (111), (200), (220), and (311) for both the as-synthesized bAgNPs confirming face-centered cubic (fcc) crystalline elemental silver. The obtained results match the JCPDS (Joint Commission of Powder Diffraction Standards) database bearing file no. (04-0783). Furthermore, the crystalline grain size of the as-synthesized bAgNPs was calculated using Scherer's equation: *D* = (*K*λ/βcosθ) [where *D* is the mean crystalline size of the particle, *K* is the shape factor whose value is 0.9, λ is the wavelength of the X-ray radiation (i.e., 0.154 nm), β is (π/180)^*^FWMH, and θ is the Bragg angle]. The average size obtained using Scherer's equation was 23.62 and 23.81 nm for Aq-bAgNPs and Et-bAgNPs, respectively (Chauhan and Chauhan, 2014; Nayak et al., 2016b).

### Investigation of Antimicrobial Activity

#### MIC and ZOI

Broth dilution method was used to determine the MIC value of the as-synthesized Aq-bAgNPs and Et-bAgNPs. In case of Aq-bAgNPs, the lowest MIC value was 0.125 μg/ml against *E. coli* DH5α, *S. typhi, V. cholerae, E coli* K12, and *H. alvei*. On the other hand, the lowest MIC value for Et-bAgNPs was also 0.125 μg/ml against *B. subtilis, V. cholerae, H. alvei*, and *A. baumannii* (Table 1). A clear zone surrounding the metrical filter paper disk confirmed the antibacterial/bactericidal activity of the as-synthesized bAgNPs. The highest antibacterial activity of Aq-bAgNPs and Et-bAgNPs was observed against *S. aureus*, a Gram-positive bacteria, and the ZOI was ~25 and 28 mm in diameter, respectively (Table 1). However, the lowest antibacterial activity of Aq-bAgNPs and Et-bAgNPs was observed against enteropathogenic *E. coli* (i.e., ~12.25 mm) and *B. subtilis* (i.e., ~11.75 mm), respectively. Aqueous stem extract, ethanolic stem extract, and silver nitrate were used as control and they showed no significant antibacterial activity against any of the tested microorganisms (Table 1). The time-dependent (i.e., 12, 16, 20, and 24 h) as well as dose-dependent (i.e., 20, 40, and 60 μg) antimicrobial activity of our as-synthesized bAgNPs is shown in Table S2. The diameter of clear zone (millimeter) surrounding the disk reduced after 16 h of incubation with bAgNPs, plant extracts, and AgNO_3_ in case of all the bacteria. The maximum diameter of clear zone (millimeter) surrounding the disk was obtained at 60 μg of bAgNPs, plant extracts, and AgNO_3_ in case of all the bacteria, which is larger than the area of clear zone for already reported biogenic silver nanoparticles (Ahmed et al., 2016; Masum et al., 2019).

**Table 1:** Zone of inhibition and MIC value for bAgNPs against bacteria.

	**MIC (μg)**		**Zone of inhibition (mm)**					**Pathogenicity**
	**Aq-bAgNPs**	**Et-bAgNPs**	**Aq-bAgNPs (60 μg)**	**Aqueous plant extract (60 μg)**	**Et-bAgNPs (60 μg)**	**Ethanolic plant extract (60 μg)**	**AgNO_**3**_**	
*B. subtilis*	0.250	0.125	17.5 ± 1.0	8.5 ± 0.0	11.8 ± 0.3	7.5 ± 0.00	7.5 ± 0.0	Nonpathogenic
*E. coli DH5α*	0.125	0.250	14.0 ± 0.5	9.5 ± 0.0	16.5 ± 0.0	7.5 ± 0.0	8.3 ± 1.3	Nonpathogenic
EPEC	0.25	0.250	12.3 ± 0.8	8.0 ± 0.0	12.8 ± 0.3	7.0 ± 0.0	7.0 ± 0.0	Pathogenic
*S. typhi*	0.125	0.250	17.8 ± 0.8	11.0 ± 0.0	15.5 ± 1.0	10 ± 0.00	7.0 ± 0.0	Pathogenic
*S. aureus*	0.250	0.250	25.0 ± 0.5	8.0 ± 0.0	28.0 ± 0.0	11.5 ± 0.0	10.3 ± 0.3	Pathogenic
*V. cholera*	0.125	0.125	17.0 ± 0.0	7.0 ± 0.0	17.5 ± 0.0	7.0 ± 0.0	8.0 ± 0.0	Pathogenic
*E. coli K12*	0.125	0.25	15.0 ± 0.0	8.0 ± 0.0	16.0 ± 0.0	8.0 ± 00	9.0 ± 0.0	Nonpathogenic
*E. faecalis*	0.250	0.250	15.8 ± 0.3	8.5 ± 0.0	15.8 ± 0.3	7.0 ± 0.00	7.0 ± 0.00	Pathogenic
*H. alvei*	0.125	0.125	15.0 ± 0.0	7.5 ± 0.0	15.3 ± 0.3	7.0 ± 0.0	7.0 ± 0.0	Pathogenic
*A. baumannii*	0.250	0.125	17.8 ± 0.3	7.0 ± 0.0	18.3 ± 0.3	7.5 ± 0.00	11.0 ± 0.0	Pathogenic

The phytoconstituents present in *A. paniculata* stem extracts contain various bioactive compounds with long hydrocarbon chain and hydroxyl as well as carboxyl groups that act as the hydrophilic moieties. Hence, the interactions between the bAgNPs and bacteria were mainly through noncovalent (i.e., hydrophobic) interactions. Therefore, the increased uptake of negatively charged Aq-bAgNPs and Et-bAgNPs was due to their higher hydrophobicity compared to plant extract and silver nitrate (Bu et al., 2010; Li et al., 2014). Secondly, the interaction between the bAgNPs and bacteria could also be due to molecular crowding (Arakha et al., 2015). Thirdly, the basic antibacterial mechanism of bAgNPs has been shown to be either due to the release of silver ions or intracellular deposition of bAgNPs (Kim et al., 2016; Hoseinnejad et al., 2018). The detailed mechanism involves the damage of cell membrane, disruption of energy metabolism, generation of oxidative stress due to reactive oxygen species (ROS) generation, and inhibition of gene transcription (Hindi et al., 2009). Silver ions released from bAgNPs interact with sulfur- and phosphorus-containing proteins present in the plasma membrane as well as in the bacterial cell wall through electrostatic interactions (Hindi et al., 2009). This leads to the pore formation in the bacterial cell membrane that executes the outflow of bacterial intracellular contents. As a result, an electrochemical imbalance is generated in the cells resulting in permanent cell damage (Dakal et al., 2016). Both the Aq-bAgNPs and Et-bAgNPs showed better antibacterial activity to Gram-positive bacteria (i.e., *S. aureus*) when compared to Gram-negative bacteria (i.e., EPEC) because the cell wall of Gram-positive bacteria contains a thick layer of peptidoglycan and abundant pores through which external molecules can enter into the cell that brings about membrane damage and cellular death (Bu et al., 2010). The difference of antibacterial activity of bAgNPs against particular bacterial strain could also be different due to the differences in lipid composition, gross composition of the membranes, or even specific protein complexes present in the surface of the bacterial cell wall (Hayden et al., 2012).

#### CellTox^**TM**^ Green Assay

**Figure 4:**
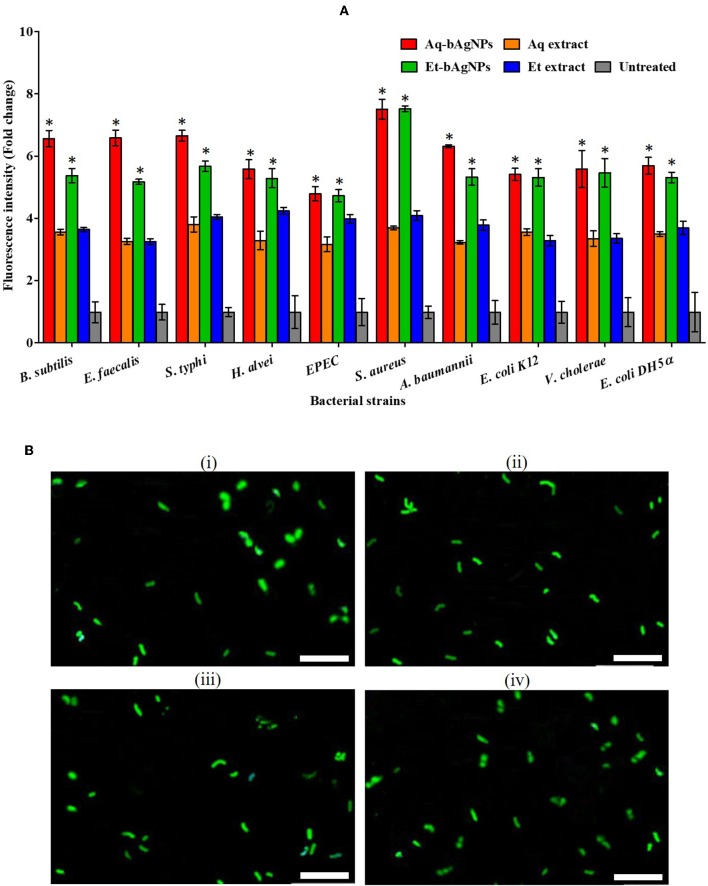
**(A)** CellTox^TM^ Green uptake assay. The fluorescence intensity of bacteria treated with Aq-bAgNPs and Et-bAgNPs was measured at 490 nm using a spectrofluorometer. The values presented are mean ± SE of multiple samples (*n* = 3). Data were analyzed using one-way ANOVA followed by Tukey's multiple comparison test. The fluorescence intensity of both bAgNP-treated bacteria was significantly higher than that of the plant extract treated as well as untreated bacteria and *P < 0.05. **(B)** CellTox^TM^ Green uptake assay. *B. subtilis* was first treated with Aq-bAgNPs (i) and Et-bAgNPs (ii). The treated bacteria were then incubated with CellTox^TM^ green to stain the cell wall compromised bacterial DNA, and green fluorescence was observed under a fluorescence microscope. The same experiment was also performed for *E. faecalis* after treating them with Aq-bAgNPs (iii) and Et-bAgNPs (iv). Scale bar: 20 μm.

CellTox^TM^ Green is a DNA binding dye. It binds to DNA of cell wall compromised bacteria and emits green fluorescence. Since CellTox^TM^ Green is impermeable to intact cell membrane, it is usually used to detect dead cells only. The green fluorescence of CellTox^TM^ Green-treated dead bacteria was quantified using a spectrofluorophotometer (SHIMADZU RF-6000, Japan) at 490 nm. The Et-bAgNP-treated *S. aureus* showed the highest fluorescence intensity followed by Aq-bAgNP-treated *S. aureus* (Figure 4A), and the intensities were ~6.5-fold higher than that of the untreated cells. The data support the ZOI (in millimeters) obtained from the disk diffusion assay. The green fluorescence of bAgNP-treated dead bacteria was also observed under a fluorescence microscope (Figure 4B and Figures S2, S3). The fluorescence images confirm the damage of bacterial cell membrane after treating them with bAgNPs and the dead bacteria appeared green.

#### Trypan Blue Dye Exclusion Assay

Trypan blue dye exclusion assay was performed to confirm the damage of bacterial cell wall because of their interactions with bAgNPs. The interaction between bAgNPs and the bacterial cell wall took place through hydrophobic interactions that resulted in damage on the cell wall. This paves the way for the entry of trypan blue dye into the bacterial cytosol from their surroundings. Consequently, the cell wall compromised or nonviable bacteria appeared blue under the phase contrast microscope (Figures S4–S8) (Ranjan Sarker et al., 2019).

### LPO Assay

**Figure 5:**
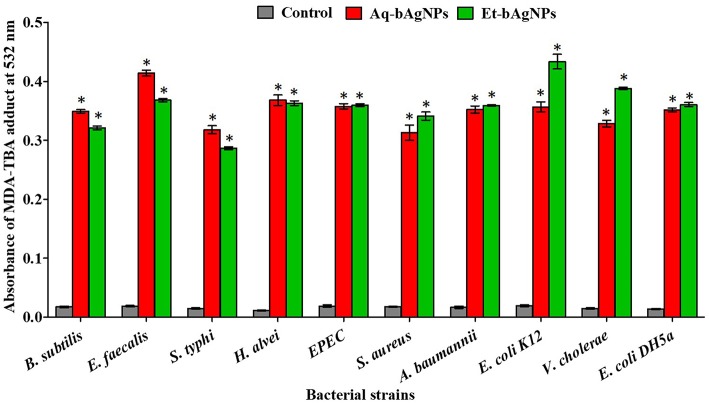
Lipid peroxidation assay. The cell membrane fatty acid oxidation potential of biogenic silver nanoparticles was measured through MDA–TBA adduct assay. The absorbance of MDA–TBA pink adduct was measured at 532 nm (i.e., λ_max_). The values presented are mean ± SE of multiple samples (*n* = 3). Data were analyzed using one-way ANOVA followed by Tukey's multiple comparison test. The absorbance of MDA–TBA adduct of bAgNPs treated bacteria were significantly higher than that of the untreated bacteria (i.e., control) and *P < 0.01.

LPO assay was performed to investigate the oxidation potential of bacterial cell membrane fatty acids by bAgNPs. The malondialdehyde–thiobarbituric acid (MDA–TBA) adduct was formed when bacteria were treated with both the bAgNPs (i.e., Aq-bAgNPs and Et-bAgNPs) because of the strong hydrophobic interactions between bAgNPs and bacterial cell wall. Among all the bacterial strains, the highest MDA–TBA adduct was formed when *E. coli* K12, a Gram-negative bacterium, was treated with Et-bAgNPs (Figure 5). However, *E. faecalis*, a Gram-positive bacterium, showed the highest amount of MDA–TBA adduct among all the bacterial strains treated with Aq-bAgNPs (Figure 5). The amount of MDA adduct is different for different bacterial strains, and it is due to the differences in the interaction of bAgNPs with bacteria. The cell wall composition of bacteria differs from species to species. The generation of lipid peroxide (LPO) took place due to the oxidation of bacterial cell membrane fatty acids by bAgNPs.

The generation of ROS is stimulated after exposure of bacterial cell membrane to bAgNPs since transition metals put oxidative stress on fatty acids (Li et al., 2012; Wang et al., 2017). The ROS oxidize the bacterial membrane fatty acids to produce lipid peroxides and the redox balance of cells favor oxidation (Wang et al., 2017). Furthermore, ROS-mediated oxidative stress inhibits the electron transport chain and alters bacterial metabolic reactions (Wang et al., 2017). Thus, it stimulates the expression of apoptotic genes and the oxidative proteins to bring about the apoptosis of the respective bacterial cell (Wang et al., 2017).

### Hemocompatibility Assay

**Figure 6:**
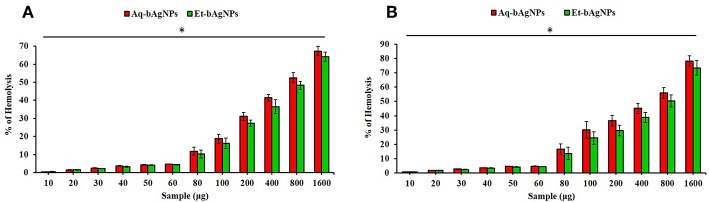
Hemocompatibility of bAgNPs to human **(A)** and rat **(B)** red blood cells (RBCs). The HC_50_ values of Aq-bAgNPs and Et-bAgNPs to human RBCs were 700 and 800 μg, respectively. However, the HC_50_ values of Aq-bAgNPs and Et-bAgNPs to rat RBCs were 600 and 800 μg, respectively. The hemocompatibility assay was performed three times on three different days. The values presented are mean ± SE of multiple samples (*n* = 3) and paired *t-*test was performed for analyzing significant difference between Aq-bAgNPs and Et-bAgNPs. The hemocompatibility of bAgNPs was significantly different from each other and *P ≤ 0.05.

Hemolytic potential assay was performed using human as well as rat RBCs to investigate the hemocompatibility of the as-synthesized bAgNPs. Both the bAgNPs showed excellent hemocompatibility to human as well as rat RBCs (Figure 6). Hemocompatibility of bAgNPs to human and rat RBCs was performed using different amount (i.e., 10–1,600 μg) of both the nanoparticles (i.e., Aq-bAgNPs and Et-bAgNPs). The HC_50_ values (the amount of nanoparticles required to lyse the 50% of RBCs) of Aq-bAgNPs and Et-bAgNPs to human RBCs were 700 and 800 μg, respectively. However, the HC_50_ values of Aq-bAgNPs and Et-bAgNPs to rat RBCs were 600 and 800 μg, respectively. The HC_50_ values are more than 10 times higher than that of the amount used for the antibacterial activity assay (i.e., 60 μg) (Figure 6). The percentage of hemolysis for 60 μg Et-bAgNPs was 4.33 and 4.35% in the case of human and rat RBCs, respectively. On the other hand, the percentage of hemolysis for 60 μg Aq-bAgNPs was 4.56 and 4.66% in the case of human and rat RBCs, respectively. The slightly higher hemocompatibility of Et-bAgNPs against both human and rat RBCs is due to their greater negative zeta potential (i.e., −26 mV) that results in stronger electrostatic repulsion with the negatively charged RBCs. These low percentage of hemolytic potential is less than that of the acceptable hemolytic value (i.e., 5%), a value that is regarded as critically safe for therapeutic applications of the biomaterials (Sarika et al., 2015; Nayak et al., 2016a). The membrane composition of rat RBCs differ significantly from that of the human RBCs (Da Silveiracavalcante et al., 2015). Therefore, the hemocompatibility of bAgNPs to human and rat RBCs is different (Da Silveiracavalcante et al., 2015).

#### Evaluation of Rat Liver and Kidney Function Biomarkers

**Figure 7:**
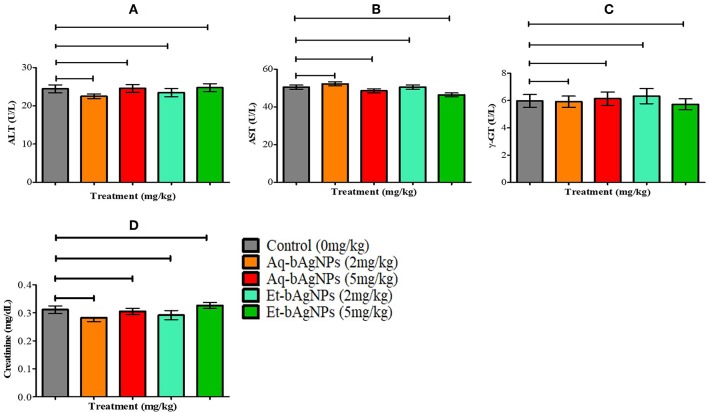
*In vivo* cytotoxicity assay. The effect of bAgNPs on liver function was investigated by measuring the levels of ALT **(A)**, AST **(B)**, and γ-GT **(C)**. The effect of bAgNPs on kidney function was also investigated by measuring the level of serum creatinine **(D)**. The values presented are mean ± SE of multiple samples (six animals per group). Data were analyzed by GraphPad Prism 5.0 (GraphPad software) using one-way ANOVA test. Dunnett test was used for *post hoc* comparison. No significant difference was observed when treatment groups were compared with control and *P* > 0.05.

The bAgNPs (i.e., Aq-bAgNPs and Et-bAgNPs) administered through intravenous route had no significant toxic effect on rat liver and kidneys (Figure 7). More specifically, there was no significant difference (*P* > 0.05) of serum ALT, AST, γ-GT, and creatinine level between the control and experimental rats (Figure 7). The data showed that neither Aq-bAgNP-treated (2 and 5 mg/kg) nor Et-bAgNP-treated (2 and 5 mg/kg) rat groups had significant influence on the aforementioned biomarkers. Liver is a vital organ of rat and colloidal AgNPs usually get deposited into it (Kim et al., 2007; Ahmad and Zhou, 2017). To determine the toxic effects of bAgNPs, liver function was investigated by measuring the level of serum ALT, AST, and γ-GT enzymes after intravenous delivery of bAgNPs. The level of ATP, a high-energy phosphate compound, decreases whenever there is any damage in liver. The decreased ATP level provokes the release of enzymes (ALT and AST) in the tissues. Thus, the level of enzymes increase in the serum whenever the liver is damaged (Gupta and Goad, 2000; Ramaiah, 2007). Gamma glutamyltransferase (γ-GT) is an enzyme of the hepatobiliary origin and its activity is minimal in normal hepatic tissues. However, the level of γ-GT rises within hours of cholestasis (impairment of bile flow) by an unknown mechanism (Ramaiah, 2007). Therefore, our data suggest that any dose of bAgNPs (up to 5 mg/kg) is therapeutically safe for liver, since no statistically significant change in the biomarkers was observed. On the other hand, the toxic effect of bAgNPs on kidneys was assessed by determining the level of serum creatinine. The low level of serum creatinine indicates the inability of kidneys to filter the waste products from the blood and excrete them through the urine (Kaur et al., 2012). Intravenous administration of different doses (up to 5 mg/kg) of bAgNPs for 7 days showed no statistically significant difference in the level of serum creatinine. Hence, bAgNPs did not show any toxic effect to kidneys besides their compatibility to liver.

## Conclusion

We synthesized biogenic silver nanoparticles (bAgNPs) in a cost-effective and ecofriendly manner. The aqueous as well as ethanolic extracts of *A. paniculata* stem were used to synthesize Aq-bAgNPs and Et-bAgNPs, respectively. The synthesis of bAgNPs was confirmed through UV-Vis, FTIR, and EDS spectroscopy, and powder X-ray diffractometer. The as-synthesized bAgNPs were characterized in terms of their size, zeta potential, shape, and morphology. The antimicrobial activity of bAgNPs was investigated against seven pathogenic and three nonpathogenic bacterial strains. Both Et-bAgNPs and Aq-bAgNPs showed the highest antimicrobial activity against pathogenic *S. aureus*, a Gram-positive bacterium. They showed the antibacterial activity through damaging the bacterial cell wall, which was confirmed through CellTox^TM^ Green assay and trypan blue dye exclusion assay. After damaging the bacterial cell wall, bAgNPs oxidize the cell membrane fatty acids to produce lipid peroxides that facilitate the interaction bAgNPs with DNA and other cellular macromolecules to bring about bacterial death. On the other hand, our as-synthesized bAgNPs showed excellent hemocompatibility and did not show any significant toxicity to liver and kidneys at a high dose. Therefore, it can be concluded that our as-synthesized bAgNPs are cost effective, have broad-spectrum antibacterial propensity and excellent biocompatibility, and can be recommended for future therapeutic applications.

## Data Availability Statement

All datasets generated for this study are included in the manuscript/Supplementary Material.

## Ethics Statement

The animal study was reviewed and approved by Biosafety, Biosecurity, and Ethical Committee of Jahangirnagar University, Dhaka, Bangladesh.

## Author Contributions

All authors listed have made a substantial, direct and intellectual contribution to the work, and approved it for publication.

### Conflict of Interest

The authors declare that the research was conducted in the absence of any commercial or financial relationships that could be construed as a potential conflict of interest.
