# Supplementary material for: Investigation of the Antibacterial Activity and in vivo Cytotoxicity of Biogenic Silver Nanoparticles as Potent Therapeutics
Source: Front Bioeng Biotechnol. 2019 Oct 9;7:239. doi: 10.3389/fbioe.2019.00239 (PMC6794407; doi:10.3389/fbioe.2019.00239)
Supplement: Supplementary file 1 [file Data_Sheet_1.pdf]

Supplementary Information

TABLE S1. Hydrodynamic size and zeta potential of biogenic AgNPs

TABLE S2: Zone of inhibition in different doses of bAgNPs and at different time points.

FIGURE S1: FTIR spectra of aqueous plant extract, ethanolic plant extract, Aq-bAgNPs, Et-bAgNPs, and silver nitrate.

FIGURE S2. CellTox™ uptake green assay shows that Aq-bAgNPs (a and b) and Et-bAgNPs (c and d) bring about bacterial (*B. subtilis*) cell wall damage. Figure b and d are bright filed images. Scale bar: 20 μm.

FIGURE S3. CellTox™ uptake green assay shows that Aq-bAgNPs (a and b) and Et-bAgNPs (c and d) bring about bacterial (*E. faecalis*) cell wall damage. Figure b and d are bright filed images. Scale bar: 20 μm.

FIGURE S4: Trypan blue dye exclusion assay to investigate the bactericidal effect of biogenic AgNPs against *B. subtilis* using Aq-bAgNPs (a), and Et-bAgNPs (b). The same experiment was also performed against *E. faecalis* using Aq-bAgNPs (c), and Et-bAgNPs (d). Dead and live cells have been indicated with black edged and white edged arrows, respectively. The images were taken using a Phase Contrast Light Microscope at (40 x 1.25)X magnification.

FIGURE S5. Trypan blue dye exclusion assay to investigate the bactericidal effect of biogenic AgNPs against *S. typhi* using Aq-bAgNPs (a) and Et-bAgNPs (b). The same experiment was also performed against *H. alvei* using Aq-bAgNPs (c) and Et-bAgNPs (d). Dead and live cells have been indicated with black edged and white edged arrows, respectively. The images were taken using a Phase Contrast Light Microscope at (40 x 1.25)X magnification.

FIGURE S6. Trypan blue dye exclusion assay to investigate the bactericidal effect of biogenic AgNPs against Enteropathogenic *E. coli* (EPEC) using Aq-bAgNPs (a) and Et-bAgNPs (b). The same experiment was also performed against *S. aureus* using Aq-bAgNPs (c) and Et-bAgNPs (d). Dead and live cells have been indicated with black edged and white edged arrows, respectively. The images were taken using a Phase Contrast Light Microscope at (40 x 1.25)X magnification.

FIGURE S7. Trypan blue dye exclusion assay to investigate the bactericidal effect of biogenic AgNPs against *A. baumannii* using Aq-bAgNPs (a) and Et-bAgNPs (b). The same experiment was also performed against *E. coli* K12 using Aq-bAgNPs (c) and Et-bAgNPs. Dead and live cells have been indicated with black edged and white edged arrows, respectively. The images were taken using a Phase Contrast Light Microscope at (40 x 1.25)X magnification.

FIGURE S8. Trypan blue dye exclusion assay to investigate the bactericidal effect of biogenic AgNPs against *V. cholera* using Aq-bAgNPs (a) and Et-bAgNPs (b). The same experiment was also performed against *E. coli* DH5α using Aq-bAgNPs (c) and Et-bAgNPs. Dead and live cells have been indicated with black edged and white edged arrows, respectively. The images were taken using a Phase Contrast Light Microscope at (40 x 1.25)X magnification.

Biochemical Assays of the collected serum

The biochemical parameters were determined in rat serum for evaluation of the toxicity in kidney and liver using a spectrophotometer (Shimadzu, Kyoto, Japan). Aspartate aminotransferase (AST) was determined through colorimetric assay according to Reitman and Frankel (Reitman and Frankel, 1957) using commercial kit. Alanine aminotransferase (ALT) was determined through Kinetic UV method according to IFCC specifications (Henley et al., 1955; Henry et al., 1960; Bergmeyer et al., 1978; Bergmeyer et al., 1986) using commercial kit. γ-glutamyltransferase (γ-GT) was determined through kinetic method described by Szasz-Persijn using Glupa-Carboxylate using commercial kit (Szasz, 1969). Serum creatinine was determined through kinetic method described by Mod. Jaffe's (Fabiny and Ertingshausen, 1971) using commercial kit. The commercial biochemical analytical kits for AST, ALT, γ-GT were purchased from Vitro Scient, Egypt and the serum creatinine determination kit was purchased from Crescent Diagnostics, Saudi Arabia.

Liver function test parameters

Estimation serum alanine transaminases (ALT) activity

The estimation of serum alanine transaminases (ALT) activity was performed according to previously established protocol (Schumann and Klauke, 2003). Briefly, serum and reagents were taken in specific tubes. They were arranged serially. Then ID number for test was entered in the analyzer. The Auto analyzer was programmed for the estimation of ALT and allowed to run with following procedure: 100 μl sample and 1000 μl reagent were mixed within the cell. The absorbance of the sample and the standard against the reagent blank were measured at 340 nm at 1, 2 and 3 minutes. The change in the absorbance per minute (ΔA/min) was determined from the linear portion of the reaction curve and the concentration of ALT was calculated by using the following formula:

$$\text{ALT activity (U/L)} = \Delta A/\text{min} \times 1746\ (30^{o}C)$$

Here, ΔA= Average absorbance of the sample in different time points (i.e. 1, 2 and 3 minutes).

Estimation of serum aspartate transaminase (AST) activity

The estimation of serum aspartate transaminases (AST) activity was performed according to previously established protocol (Schumann and Klauke, 2003). Briefly, serum and reagents were taken in specific tubes. They were arranged serially. Then ID number for test was entered in the analyzer. The Auto analyzer was programmed for the estimation of AST and allowed to run with following procedure: 100 µl sample and 1000 µl reagent were mixed within the cell. The absorbance of the sample and the standard against the reagent blank were measured at 340 nm at 1, 2 and 3 minutes. The change in absorbance per minute (ΔA/min) was determined from the linear portion of the reaction curve and the AST concentration was calculated by using the following formula:

$$\text{AST activity (U/L)} = \Delta A/\text{min} \times 1746\ (30°C)$$

Here, ΔA= Average absorbance of the sample in different time points (i.e. 1, 2 and 3 minutes).

Estimation of serum γ- glutamyltransferase (γ-GT) activity

The estimation of serum γ- glutamyltransferase (γ-GT) activity was performed according to previously established protocol (Szasz, 1969). Briefly, serum and reagents were taken in specific tubes. They were arranged serially. Then ID number for test was entered in the analyzer. The Auto analyzer was programmed for the estimation of creatinine and allowed to run with following procedure: 50 µl sample and 1000 µl reagent were mixed within the cell. The absorbance of the sample and the standard against the reagent blank were measured at 405 nm at 1, 2, and 3 minutes. The change in absorbance per minute (ΔA/min) was determined from the linear portion of the reaction curve and the γGT concentration was calculated by using the following formula:

$$\gamma\text{GT activity (U/L)} = 2210 \times \Delta A\ 405\ \text{nm/min}$$

Here, ΔA= Average absorbance of the sample in different time points (i.e. 1, 2 and 3 minutes).

One international unit (U) is defined as the amount of enzyme that catalyzes the transformation of one micromole of substrate per minute under specified conditions.

Kidney function test parameters

Estimation of serum creatinine concentration

The estimation of serum creatinine concentration was performed according to previously established protocol (Bowers and Wong, 1980). Serum and reagents were taken in specific tubes. They were arranged serially. Then ID number for test was entered in the analyzer. The Auto analyzer was programmed for the estimation of creatinine and allowed to run with following procedure: 20 µl sample or standard and 200 µl reagent were mixed within the cell. The absorbance of the sample and the standard against the reagent blank were measured at 510 nm at 30 and 90 seconds. Concentration of creatinine in the sample was calculated by using software program with the following formula:

Creatinine concentration (mg/dl) = {(A_2-A_2) sample / (A_2-A_2) standard}X C Standard

A=Absorbance of sample in different time points (i.e. 30 and 90 seconds).

C = Creatinine standard.

All experiments were carried out in triplicate and the results presented are the average measurements of the runs with standard deviation.

TABLE S1. Hydrodynamic size and zeta potential of biogenic AgNPs

**bAgNPs**	**Hydrodynamic size (nm)**	**PDI**	**Zeta potential (mV)**
Aq-bAgNPs	428 ± 197 (86.7%)<br>73 ± 19 (10.6%)	0.404	-22 ± 0.9
Et-bAgNPs	190 ± 102 (89.1%)<br>26 ± 7 (8.1%)	0.435	-26 ± 1.4

TABLE S2: Zone of inhibition in different doses of bAgNPs and at different time points

Name of strains	Hour	Zone of inhibition (mm)								
		Aqueous stem-bAgNPs			Aqueous plant extract	Ethanolic stem-bAgNPs			Ethanolic plant extract	$AgNO_3$
		60 µg	40 µg	20 µg		60 µg	40 µg	20 µg		
B. subtilis	12	17 ± 1.00	14.5 ± 0.50	13.5 ± 0 .50	8 ± 0.00	11.25 ± 0.25	10.25 ± 0.25	9.75 ± 0.75	7 ± 0.00	7 ± 0.00
	16	17.5 ± 1.00	14.25 ± 0.25	13.75 ± 0.25	8.5 ± 0.00	11.75 ± 0.25	10.75 ± 0.25	10.25 ± 0.25	7.5 ± 0.00	7.5 ± 0.00
	20	14 ± 0.50	13 ± 0.00	11 ± 1.00	7.5 ± 0.00	10.75 ± 0.25	10.25 ± 0.25	9.25 ± 0.25	6.5 ± 0.00	7 ± 0.00
	24	9.5 ± 0.50	11 ± 1.00	9.5 ± 0.50	7 ± 0.00	10.25 ± 0.25	9.75 ± 0.25	8.75 ± 0.25	6 ± 0.00	6.5 ± 0.00
E. coli DH5α	12	13.5 ± 0.50	12.5 ± 0.50	11.5 ± 0.50	9 ± 0.00	16 ± 0.00	13.5 ± 0.50	10.75 ± 0.75	8 ± 0.00	7.75 ± 1.25
	16	14 ± 0.50	13 ± 0.50	12 ± 0.50	9.5 ± 0.00	16.5 ± 0.00	14.25 ± 0.25	11.25 ± 0.75	7.5 ± 0.00	8.25 ± 1.25
	20	11.75 ± 0.25	10.5 ± 0.50	10.25 ± 0.25	7 ± 0.00	13.5 ± 0.50	12.25 ± 0.25	9.25 ± 0.25	7 ± 0.00	6.5 ± 0.5
	24	11.25 ± 0.25	9.75 ± 0.25	9.25 ± 0.25	6 ± 0.00	12.75 ± 0.25	11 ± 0.00	9 ± 0.00	6 ± 0.00	6.25 ± 0.25

Name of strains	Hour	Zone of inhibition (mm)								
		Aqueous stem-bAgNPs			Aqueous plant extract	Ethanolic stem-bAgNPs			Ethanolic plant extract	$AgNO_3$
		60 µg	40 µg	20 µg		60 µg	40 µg	20 µg		
EPEC	12	11.5 ± 0.50	10.75 ± 0.25	10.25 ± 0.25	8.5 ± 0.00	12.25 ± 0.25	11.25 ± 0.25	10.5 ± 0.50	8 ± 0.00	6.5 ±0.00
	16	12.25 ± 0.75	11.5 ± 0.50	10.75 ± 0.25	8 ± 0.00	12.75 ± 0.25	11.75 ± 0.25	11 ± 0.50	7 ± 0.00	7 ± 0.00
	20	10.75 ± 0.25	10.25 ± 0.25	9.5 ± 0.50	7 ± 0.00	11.25 ± 0.25	10.25 ± 0.25	8.75 ± 0.25	6.5 ± 0.00	6 ± 0.00
	24	10.25 ± 0.25	9.25 ± 0.25	8.5 ± 0.50	6.5 ± 0.00	10.25 ± 0.25	8.5 ± 0.5	8.25 ± 0.25	6.5 ± 0.00	6 ± 0.00
S. typhi	12	17.25 ± 0.75	16.5 ± 0.50	15.75 ± 0.25	11 ± 0.00	15 ± 1.00	13.5 ± 0.50	12.25 ± 0.25	10 ± 0.00	7 ± 0.00
	16	17.75 ± 0.75	17 ± 0.50	16 ± 0.00	11 ± 0.00	15.5 ± 1.00	14 ± 0.50	12.5 ± 0.00	10 ± 0.00	7 ± 0.00
	20	17.25 ± 0.25	15.25 ± 0.25	14.75 ± 0.25	10.5 ± 0.00	14.75 ± 1.75	12.5 ± 0.50	11.25 ± 0.25	9 ± 0.00	6.5 ± 0.00
	24	16 ± 0.50	14.75 ± 0.75	14.5 ± 0.50	10 ± 0.00	14.5 ± 1.5	12 ± 0.00	10.75 ± 0.25	8.5 ± 0.00	6.5 ± 0.00
S. aureus	12	22.5 ± 0.00	22 ± 0.00	21 ± 0.00	7.5 ± 0.00	26.25 ± 0.25	24.25 ± 0.25	23.25 ± 0.25	11 ± 0.00	10 ± 0.00
	16	25 ± 0.50	23.25 ± 0.25	22.75 ± 0.25	8 ± 0.00	28 ± 0.00	25.75 ± 0.25	24.75 ± 0.25	11.5 ± 0.00	10.25 ± 0.25
	20	20.5 ± 0.50	19.25 ± 0.25	18.75 ± 0.25	6.5 ± 0.00	22.75 ± 0.25	21.25 ± 0.25	20.25 ± 0.25	9 ± 0.00	7 ± 0.00
	24	17.25 ± 0.25	16.75 ± 0.75	15.5 ± 0.50	6 ± 0.00	19.75 ± 0.25	17.75 ± 0.25	17.25 ± 0.25	8 ± 0.00	6.5 ± 0.00
V. cholerae	12	15.5 ± 0.50	14.75 ± 0.25	13.25 ± 0.25	6.5 ± 0.00	16 ± 0.00	15 ± 0.00	14.25 ± 0.25	7 ± 0.00	7 ± 0.00
	16	17 ± 0.00	16.25 ± 0.25	15 ± 0.00	7 ± 0.00	17.5 ± 0.00	15.75 ± 0.25	15 ± 0.00	7 ± 0.00	8 ± 0.00
	20	15.25 ± 0.25	14.25 ± 0.25	13.75 ± 0.25	6 ± 0.00	15.75 ± 0.25	13.75 ± 0.25	12.25 ± 0.25	6 ± 0.00	7 ± 0.00
	24	13.75 ± 0.25	13.25 ± 0.25	11.75 ± 0.25	6 ± 0.00	14.25 ± 0.25	11.75 ± 0.25	11.25 ± 0.25	6 ± 0.00	6 ± 0.00

Name of strains	Hour	Zone of inhibition (mm)								
		Aqueous stem-bAgNPs			Aqueous plant extract	Ethanolic stem-bAgNPs			Ethanolic plant extract	$AgNO_3$
		60 µg	40 µg	20 µg		60 µg	40 µg	20 µg		
E coli K12	12	13.75 ± 0.25	13.25 ± 0.25	12.75 ± 0.25	8 ± 0.00	15.25 ± 0.25	13.25 ± 0.25	11.5 ± 1.50	8 ± 0.00	8.5 ± 0.00
	16	15 ± 0.00	14.75 ± 0.25	14 ± 0.00	8 ± 0.00	16 ± 0.00	14.75 ± 0.25	12.5 ± 1.50	8 ± 0.00	9 ± 0.00
	20	12.75 ± 0.25	12.25 ± 0.25	12.75 ± 0.25	6 ± 0.00	13.75 ± 0.25	11.75 ± 0.25	10.25 ± 0.25	7 ± 0.00	7 ± 0.00
	24	11.25 ± 0.25	10.5 ± 0.50	10.25 ± 0.25	6 ± 0.00	11.75 ± 0.25	10.25 ± 0.25	9.75 ± 0.25	6 ± 0.00	6.5 ± 0.00
E. faecalis	12	13.75 ± 0.25	13.25 ± 0.25	13 ± 0.00	8 ± 0.00	14.5 ± 0.50	13.5 ± 0.00	12.25 ± 0.25	7 ± 0.00	6.5 ± 0.00
	16	15.75 ± 0.25	14.75 ± 0.25	14.75 ± 0.25	8.5 ± 0.00	15.75 ± 0.25	14.75 ± 0.25	13.75 ± 0.25	7 ± 0.00	7 ± 0.00
	20	13.25 ± 0.25	11.75 ± 0.25	10.75 ± 0.25	6 ± 0.00	13.75 ± 0.25	12.75 ± 0.25	11.75 ± 0.25	6 ± 0.00	6 ± 0.00
	24	10.75 ± 0.25	9.75 ± 0.25	9.25 ± 0.25	6 ± 0.00	12 ± 0.00	11.25 ± 0.25	10.25 ± 0.25	6 ± 0.00	6 ± 0.00
H. alvei	12	13.25 ± 0.25	12.75 ± 0.25	12.25 ± 0.25	7 ± 0.00	14 ± 0.00	13.25 ± 0.25	12.25 ± 0.25	7 ± 0.00	6.5 ± 0.00
	16	15 ± 0.00	14.25 ± 0.25	13.25 ± 0.25	7.5 ± 0.00	15.25 ± 0.25	14.5 ± 0.25	13.5 ± 0.00	7 ± 0.00	7 ± 0.00
	20	13.25 ± 0.25	12.25 ± 0.25	11.75 ± 0.25	6 ± 0.00	13.25 ± 0.25	11.75 ± 0.25	10.75 ± 0.25	6.5 ± 0.00	6 ± 0.00
	24	11.25 ± 0.25	10.5 ± 0.50	10 ± 0.00	6 ± 0.00	11.25 ± 0.25	10.5 ± 0.50	9.25 ± 0.25	6 ± 0.00	6 ± 0.00
A. baumannii	12	16.25 ± 0.25	14.75 ± 0.25	13.75 ± 0.25	7.5 ± 0.00	17.25 ± 0.25	15.75 ± 0.25	15.25 ± 0.25	8 ± 0.00	10 ± 0.00
	16	17.75 ± 0.25	16.5 ± 0.25	15.25 ± 0.25	7 ± 0.00	18.25 ± 0.25	16.5 ± 0.25	16.25 ± 0.25	7.5 ± 0.00	11 ± 0.00
	20	15.75 ± 0.25	14.25 ± 0.25	13.75 ± 0.25	6 ± 0.00	16.25 ± 0.25	15.25 ± 0.25	14 ± 0.25	7 ± 0.00	9.5 ± 0.00
	24	13.25 ± 0.25	12.25 ± 0.25	10.25 ± 0.25	6 ± 0.00	14.25 ± 0.25	13.25 ± 0.25	12.75 ± 0.25	6.5 ± 0.00	8 ± 0.00

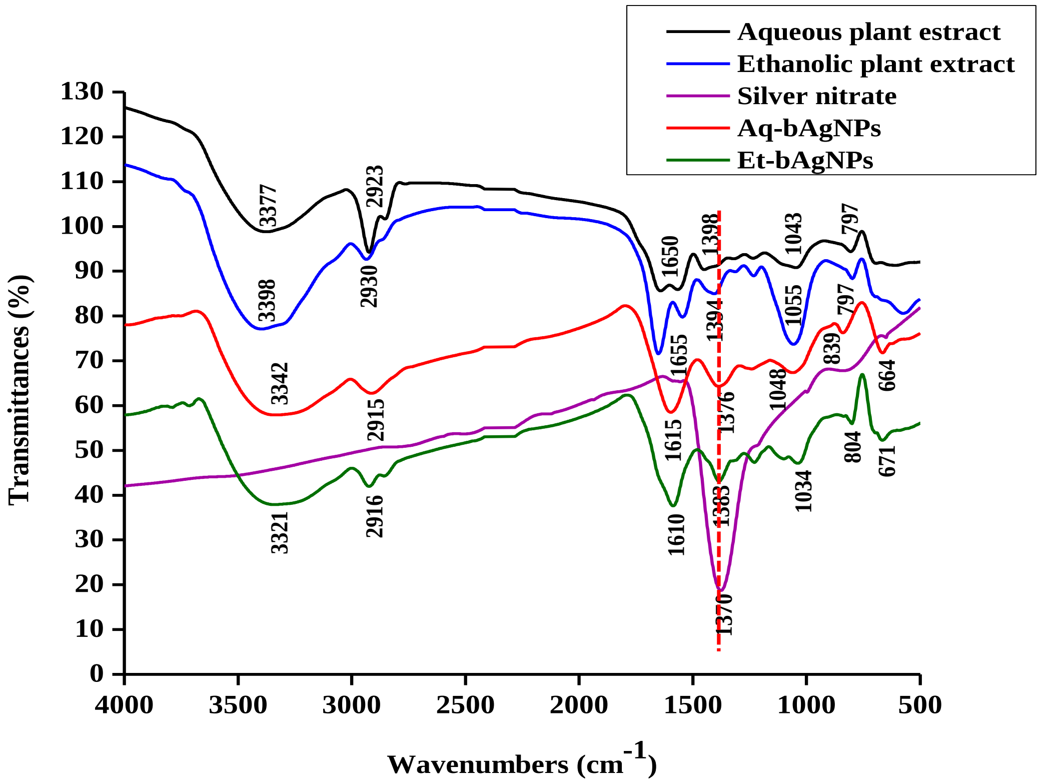

FIGURE S1: FTIR spectra of aqueous plant extract, ethanolic plant extract, Aq-bAgNPs, Et-bAgNPs, and silver nitrate.

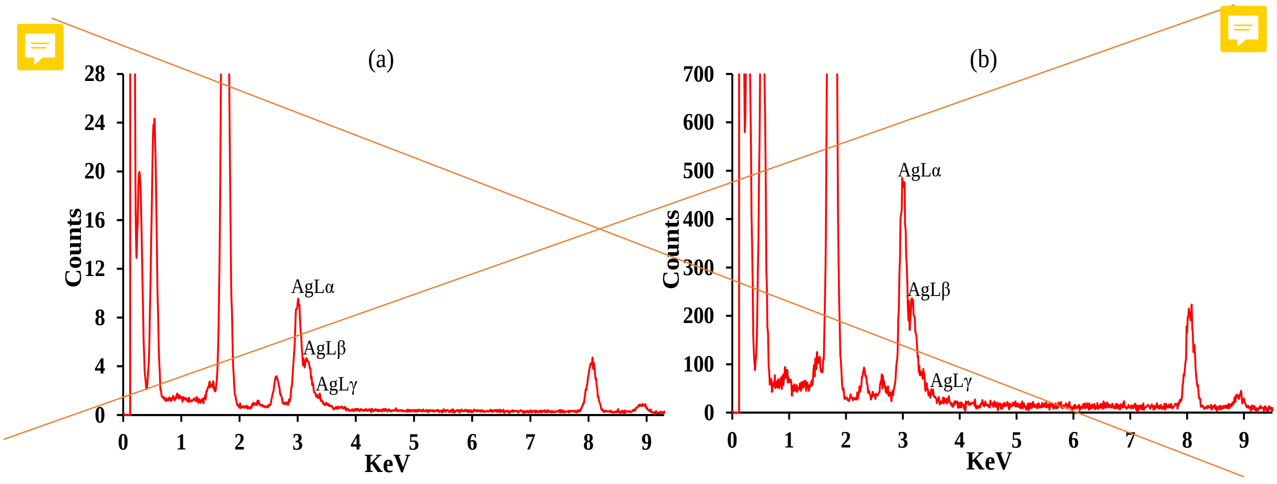

~~FIGURE S2: Energy dispersive X-ray (EDS) spectra of Aq-bAgNPs (a), and Et-bAgNPs (b). Peaks at ~3 KeV indicate the presence of Ag in the biogenic nanoparticles.~~

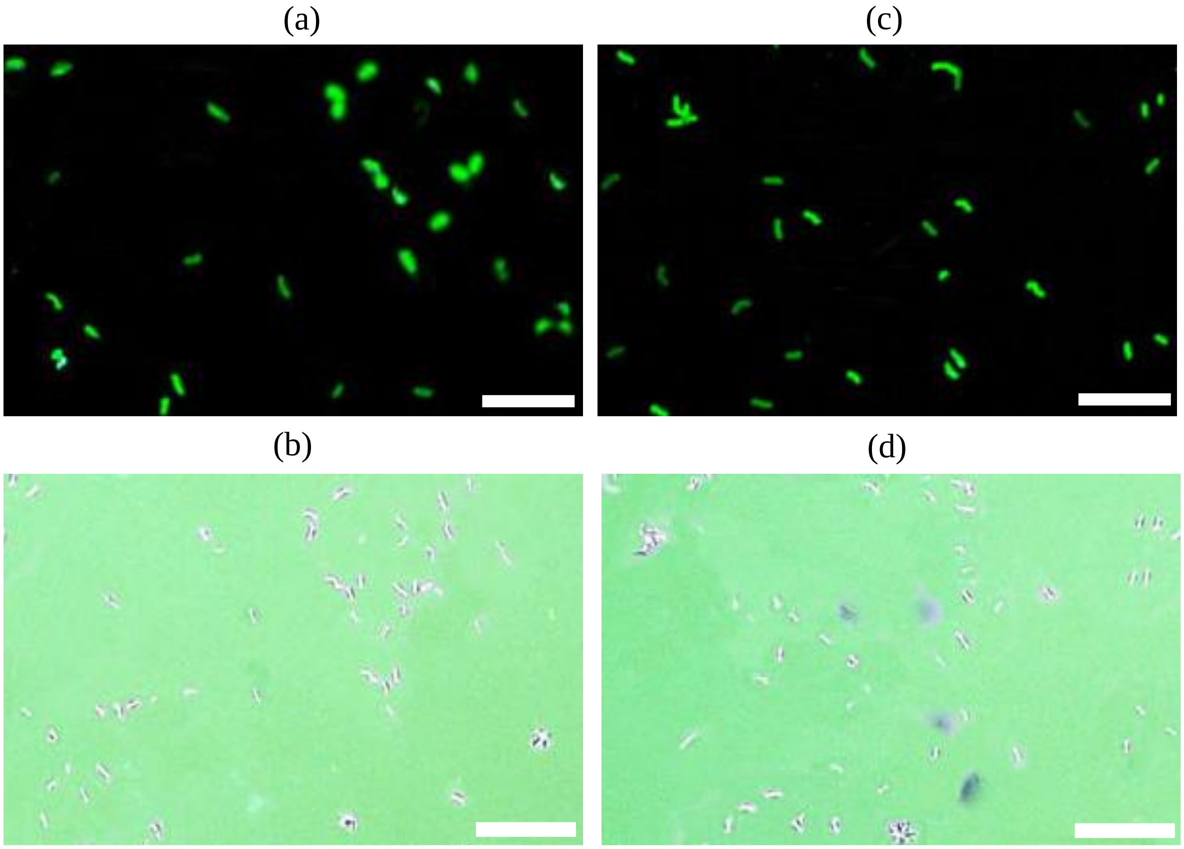

FIGURE S2. CellTox™ Green assay shows that Aq-bAgNPs (a and b) and Et-bAgNPs (c and d) bring about bacterial (*B. subtilis*) cell wall damage. Figure b and d are bright filed images. Scale bar: 20 μm.

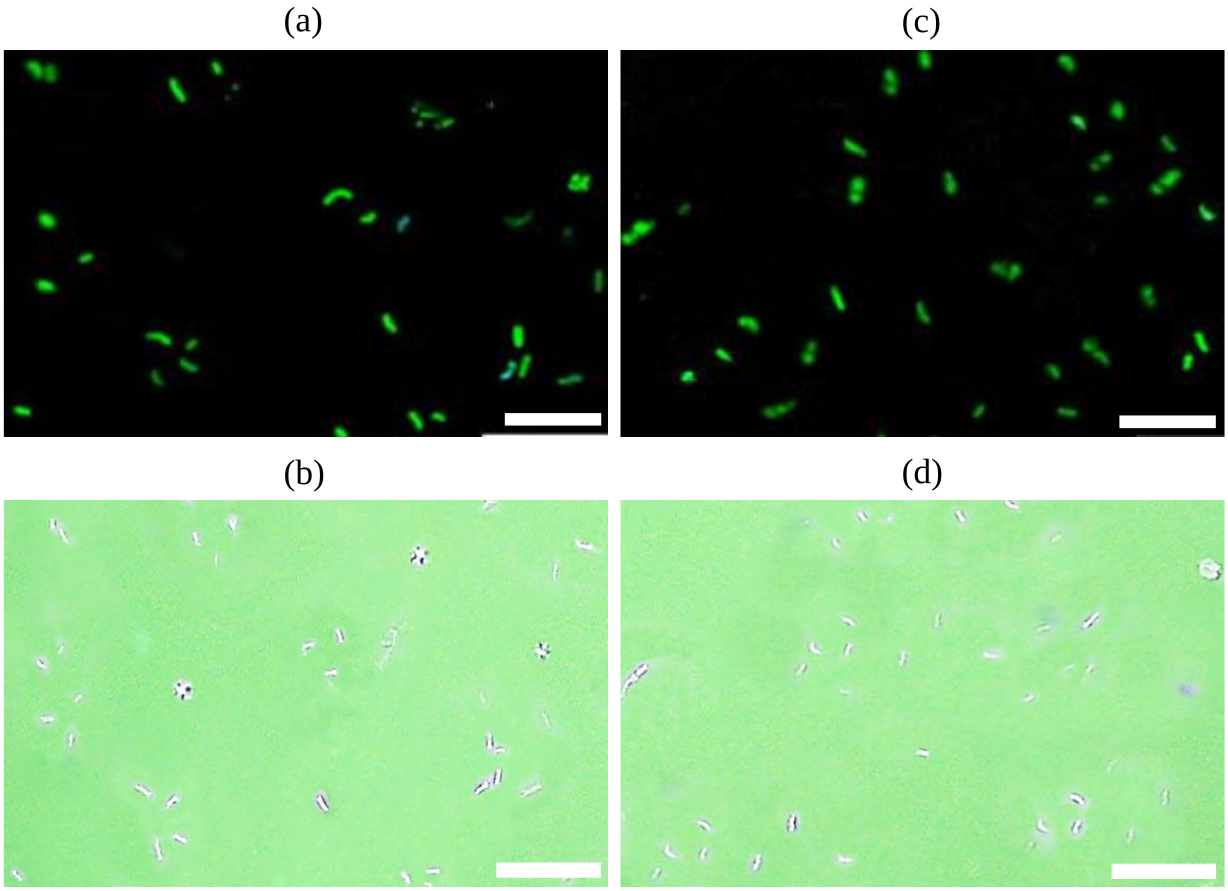

FIGURE S3. CellTox^{TM} Green assay shows that Aq-bAgNPs (a and b) and Et-bAgNPs (c and d) bring about bacterial (*E. faecalis*) cell wall damage. Figure b and d are bright filed images. Scale bar: 20 μm.

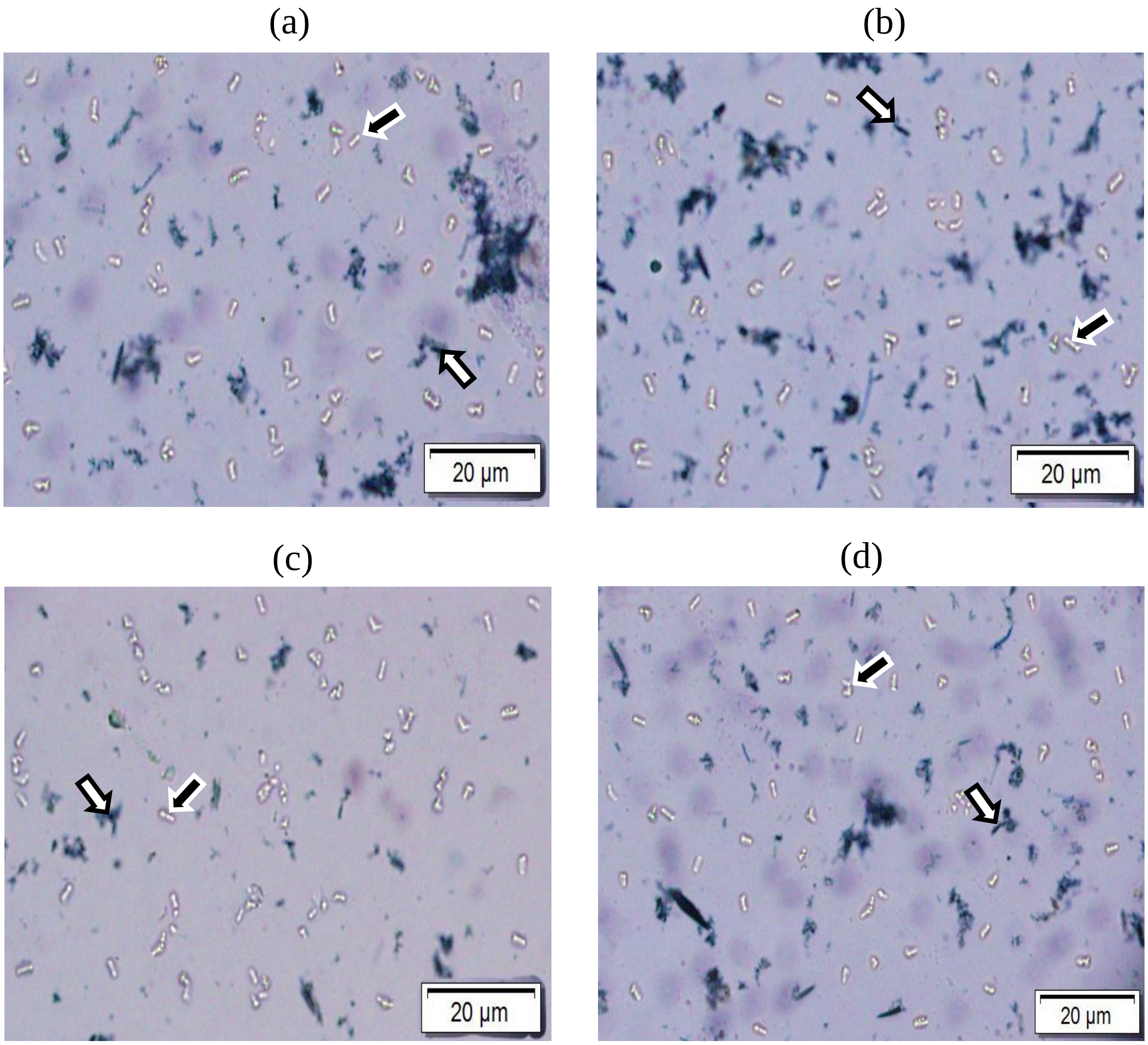

FIGURE S4: Trypan blue dye exclusion assay to investigate the bactericidal effect of biogenic AgNPs against *B. subtilis* using Aq-bAgNPs (a), and Et-bAgNPs (b). The same experiment was also performed against *E. faecalis* using Aq-bAgNPs (c), and Et-bAgNPs (d). Dead and live cells have been indicated with black edged and white edged arrows, respectively. The images were taken using a Phase Contrast Light Microscope at (40 x 1.25)X magnification.

(a) (b)

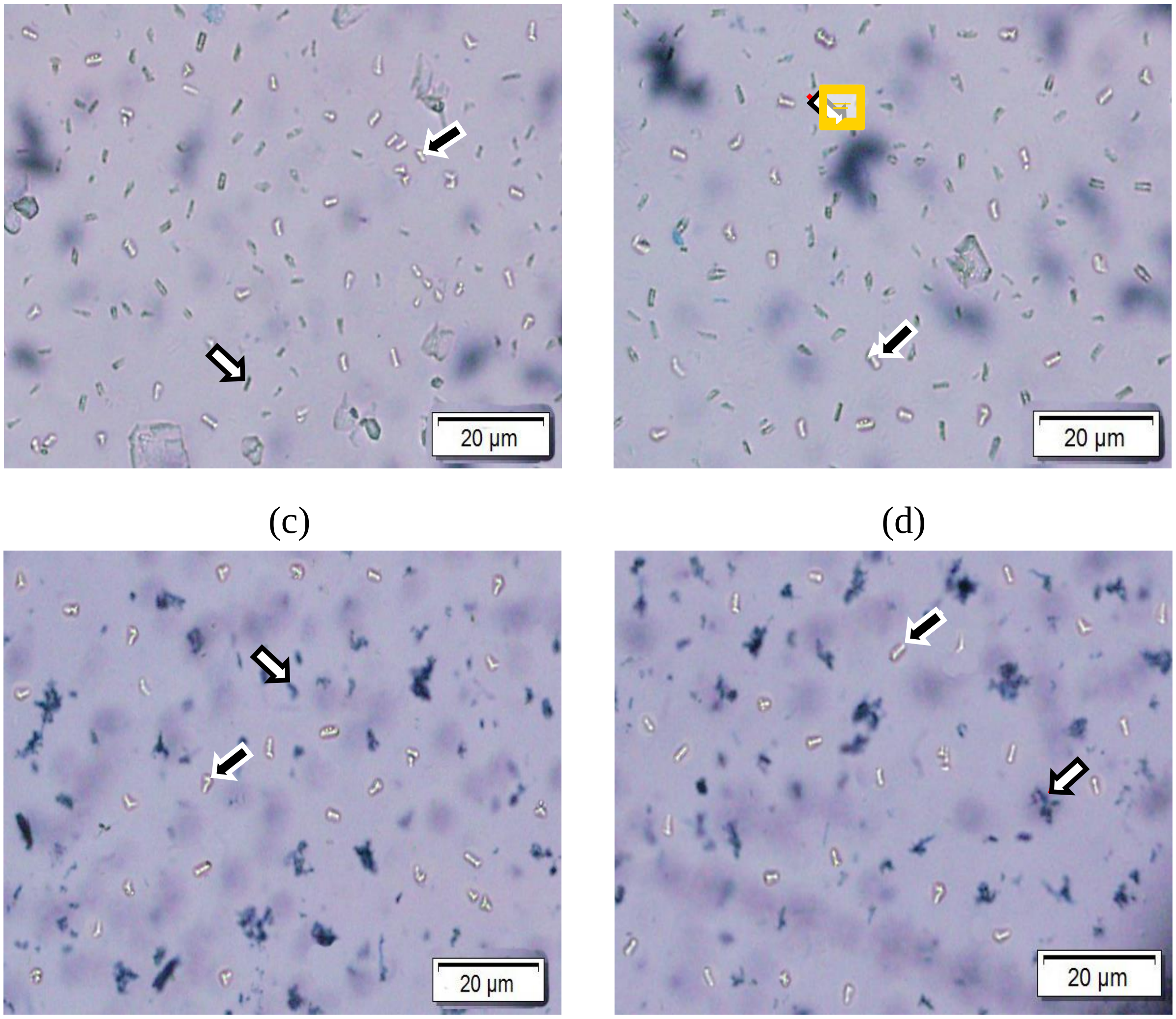

(c) (d)

FIGURE S5. Trypan blue dye exclusion assay to investigate the bactericidal effect of biogenic AgNPs against *S. typhi* using Aq-bAgNPs (a) and Et-bAgNPs (b). The same experiment was also performed against *H. alvei* using Aq-bAgNPs (c) and Et-bAgNPs (d). Dead and live cells have been indicated with black edged and white edged arrows, respectively. The images were taken using a Phase Contrast Light Microscope at (40 x 1.25)X magnification.

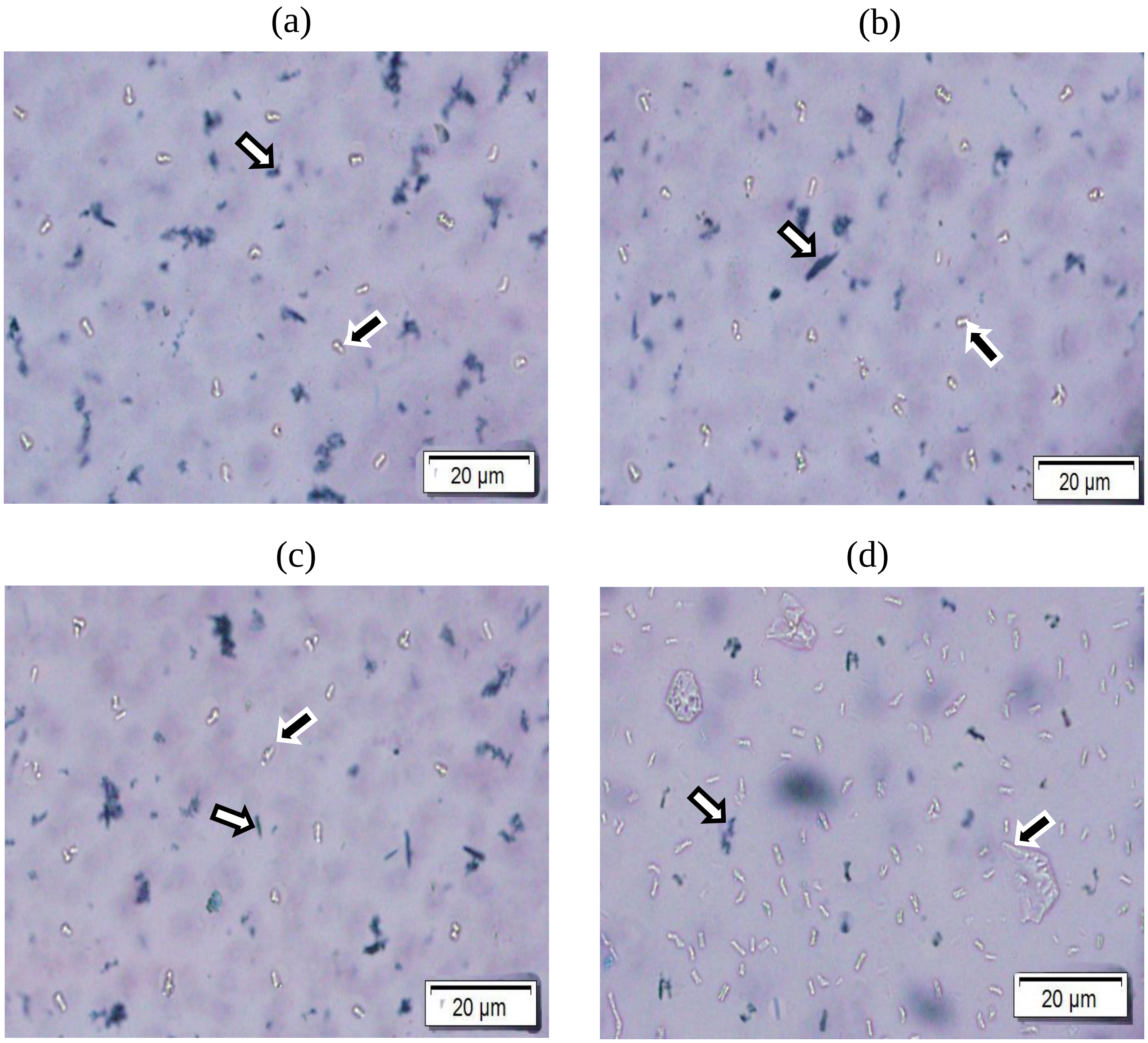

FIGURE S6. Trypan blue dye exclusion assay to investigate the bactericidal effect of biogenic AgNPs against Enteropathogenic *E. coli* (EPEC) using Aq-bAgNPs (a) and Et-bAgNPs (b). The same experiment was also performed against *S. aureus* using Aq-bAgNPs (c) and Et-bAgNPs (d). Dead and live cells have been indicated with black edged and white edged arrows, respectively. The images were taken using a Phase Contrast Light Microscope at (40 x 1.25)X magnification.

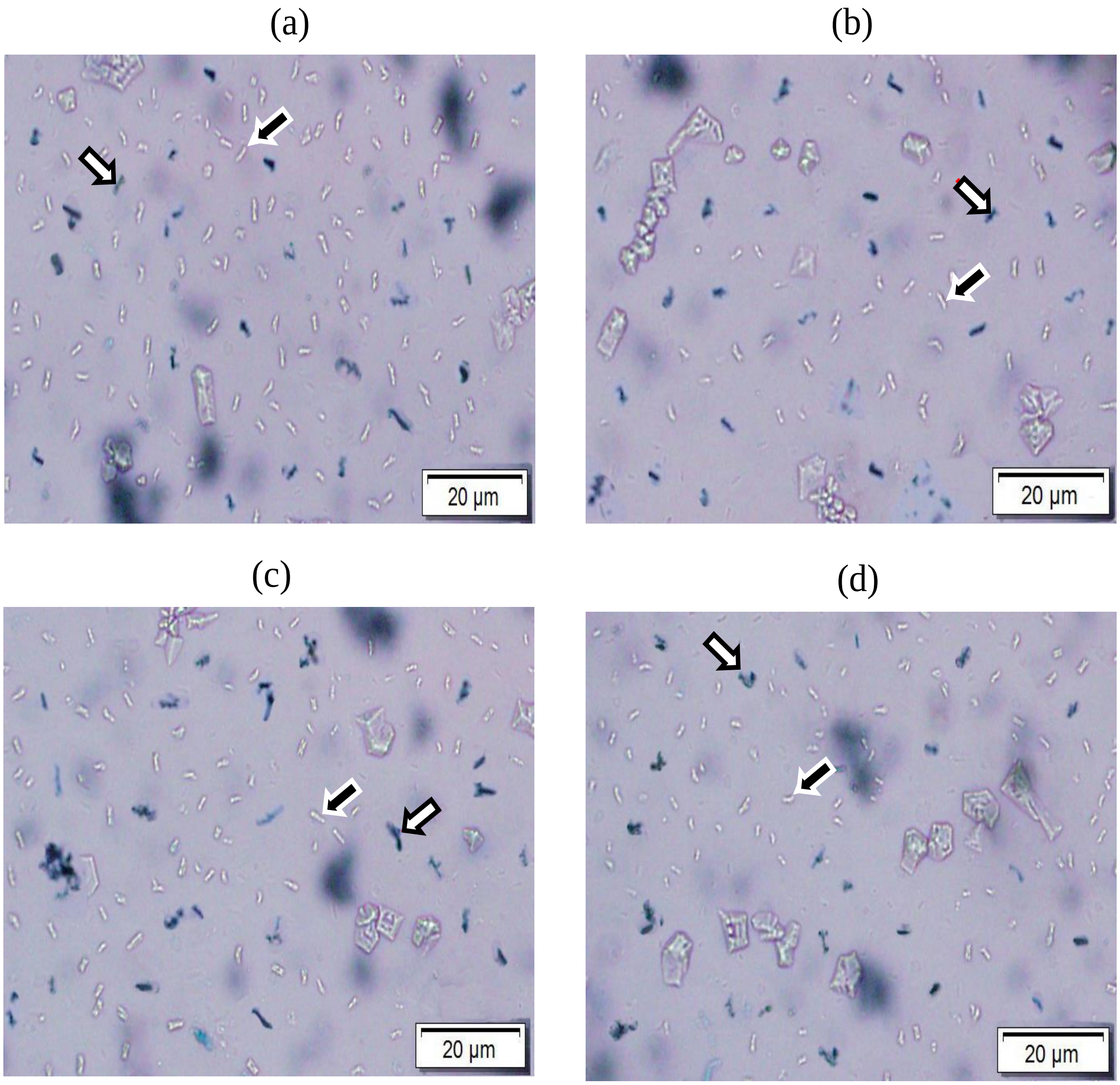

FIGURE S7. Trypan blue dye exclusion assay to investigate the bactericidal effect of biogenic AgNPs against *A. baumannii* using Aq-bAgNPs (a) and Et-bAgNPs (b). The same experiment was also performed against *E. coli* K12 using Aq-bAgNPs (c) and Et-bAgNPs. Dead and live cells have been indicated with black edged and white edged arrows, respectively. The images were taken using a Phase Contrast Light Microscope at (40 x 1.25)X magnification.

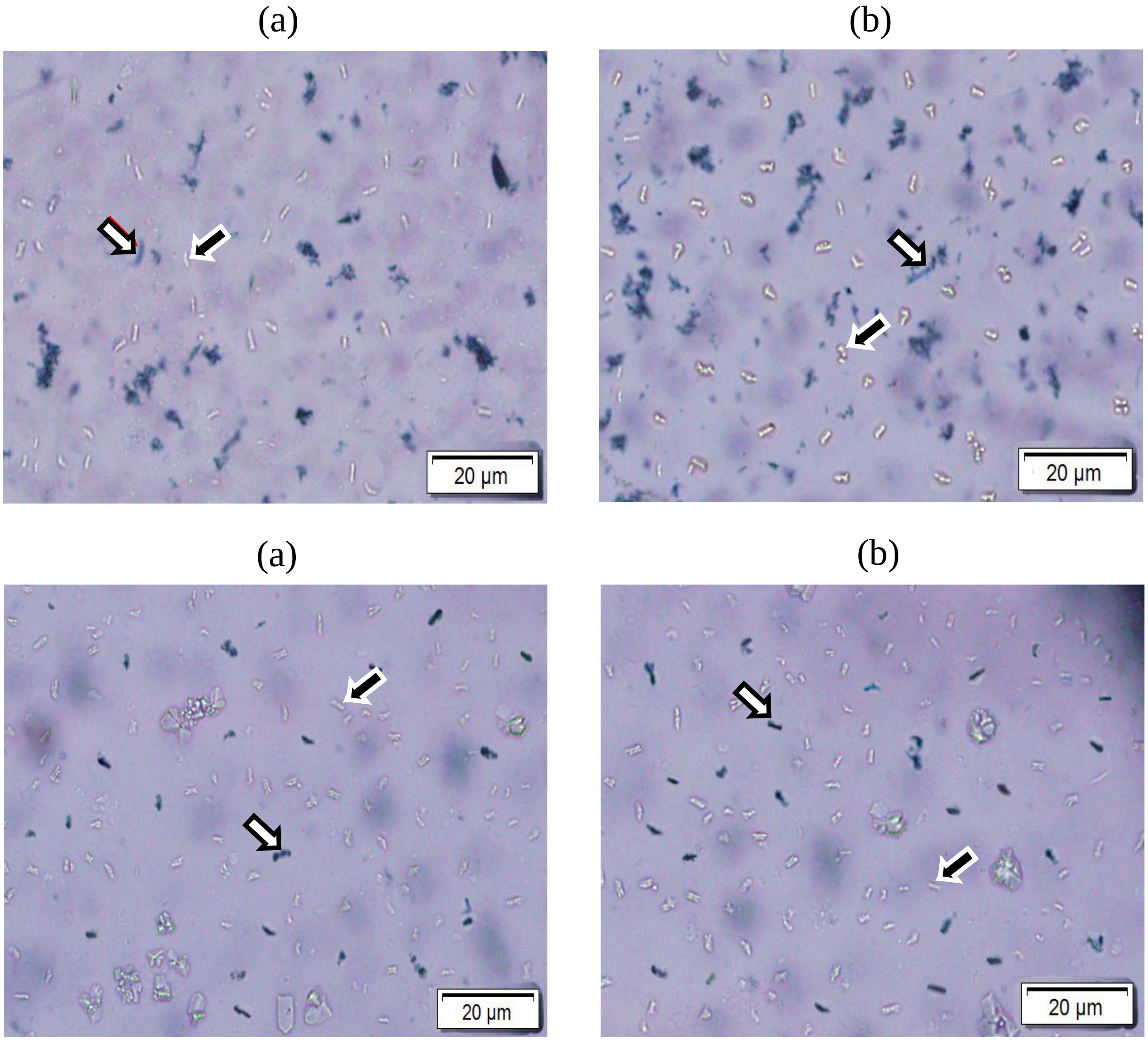

FIGURE S8. Trypan blue dye exclusion assay to investigate the bactericidal effect of biogenic AgNPs against *V. cholera* using Aq-bAgNPs (a) and Et-bAgNPs (b). The same experiment was also performed against *E. coli* DH5α using Aq-bAgNPs (c) and Et-bAgNPs. Dead and live cells have been indicated with black edged and white edged arrows, respectively. The images were taken using a Phase Contrast Light Microscope at (40 x 1.25)X magnification.

References

Bergmeyer, H., Horder, M. and Rej, R. J. J. C. C. C. B. (1986). Method for the measurement of catalytic concentration of enzymes. IFCC method for alanine aminotransferase. 24**,** 481-495.

Bergmeyer, H., Scheibe, P. and Wahlefeld, A. J. C. C. (1978). Optimization of methods for aspartate aminotransferase and alanine aminotransferase. 24**,** 58-73.

Bowers, L. D. and Wong, E. T. J. C. C. (1980). Kinetic serum creatinine assays. II. A critical evaluation and review. 26**,** 555-561.
Fabiny, D. L. and Ertingshausen, G. J. C. C. (1971). Automated reaction-rate method for determination of serum creatinine with the CentrifiChem. 17**,** 696-700.
Henley, K. S., Pollard, H. M. J. J. O. L. and Medicine, C. (1955). A new method for the determination of glutamic oxalacetic and glutamic pyruvic transaminase in plasma. 46**,** 785-789.
Henry, R. J., Chiamori, N., Golub, O. J. and Berkman, S. J. a. J. O. C. P. (1960). Revised spectrophotometric methods for the determination of glutamic-oxalacetic transaminase, glutamic-pyruvic transaminase, and lactic acid dehydrogenase. 34**,** 381-398.
Reitman, S. and Frankel, S. J. a. J. O. C. P. (1957). A colorimetric method for the determination of serum glutamic oxalacetic and glutamic pyruvic transaminases. 28**,** 56-63.
Schumann, G. and Klauke, R. J. C. C. A. (2003). New IFCC reference procedures for the determination of catalytic activity concentrations of five enzymes in serum: preliminary upper reference limits obtained in hospitalized subjects. 327**,** 69-79.
Szasz, G. J. C. C. (1969). A kinetic photometric method for serum γ-glutamyl transpeptidase. 15**,** 124-136.
